# Integrating zero-inflation correction and transcriptional kinetics for single-cell transcriptomic analysis

**DOI:** 10.1371/journal.pcbi.1014779

**Published:** 2026-09-25

**Authors:** Chengkai Yang, Yu Liao, Ying Sheng, Feng Jiao

**Affiliations:** 1 College of Mathematics and Information Sciences, Guangzhou University, Guangzhou, P. R. China; 2 Guangzhou Center for Applied Mathematics, Guangzhou University, Guangzhou, P. R. China; Jiangnan University, CHINA

## Abstract

Single-cell transcriptomic data exhibit pervasive zero inflation, while traditional models either neglect this issue or fail to capture transcriptional burst-driven bimodality, hindering accurate gene regulatory studies. This study developed a zero-inflated telegraph model that integrates technical zero correction with the stochastic gene state-switching dynamics of the classical telegraph model. Systematic validation was conducted using synthetic data, human scRNA-seq data from lupus and breast cancer patients, and mouse embryonic stem cell scRNA-seq data. The model showed superior performance: it accurately fits mRNA distributions (including bimodal patterns), reliably estimates effective transcriptional burst parameters while preventing overfitting, thus enables correction of traditional models’ regulatory inference bias. It also outperforms conventional approaches in detecting differentially expressed genes, with notable advantages in small samples, and identifies unique disease-related genes (e.g., *LDLR, GZMB* for lupus, *FAIM2, VDR* for breast cancer). This biologically interpretable and robust tool advances single-cell transcriptomic analysis.

## Author summary

Single-cell studies demonstrate that gene expression is an inherently stochastic process driven by transcriptional bursting. Genes randomly toggle between active and inactive states, causing variable mRNA abundance even among genetically identical cells. The widespread absence of transcripts in such data arises from both biological noise and sequencing dropouts, a phenomenon referred to as zero inflation. Many existing approaches struggle to simultaneously address zero inflation and burst-associated expression dynamics. To address this challenge, we present a zero-inflated telegraph model that integrates technical zero correction with stochastic gene regulatory dynamics. Tested on synthetic data, mouse and human single-cell transcriptomic datasets, our model produces reliable mRNA distribution fits, distinguishes biological signals from technical artifacts, and yields reasonable estimates of transcriptional burst parameters. It also performs well in differential gene detection, especially with limited samples. This framework may serve as a useful tool for single-cell transcriptomic analysis.

## Introduction

Gene transcription is an inherently stochastic process, a phenomenon evidenced by the temporal fluctuations in messenger RNA (mRNA) copy numbers observed even within clonally homogeneous (isogenic) cell populations  [1,2]. Quantifying mRNA molecules at the single-cell level generates large-scale datasets of mRNA copy number distributions ($P_{m}$), which explicitly quantify the probability that a single cell contains exactly *m* mRNA molecules of the gene of interest  [3,4]. When integrated with mechanistic mathematical models, these datasets provide a statistical framework for estimating key system parameters and elucidating the underlying mechanisms of stochastic gene regulation. Such analyses hold significant utility across diverse biological fields, including inferring the genetic circuits governing cell fate determination  [5,6] and cellular responses to environmental perturbations  [7,8], as well as identifying differentially expressed genes (DEGs) between control and pathogenic conditions to prioritize therapeutic targets  [9,10].

To model the stochastic nature of transcription, the traditional constitutive model is insufficient. This is because the Poisson distribution it generates cannot describe the observed distribution, where the mean deviates significantly from the distribution peak  [1]. In contrast, this phenomenon can be well captured by the classical telegraph model. It posits that a gene stochastically transitions between an active state and an inactive state; mRNA molecules are synthesized during the active state  [1,3,11]. The telegraph model can effectively characterize the unimodal and bimodal distributions observed in bacteria  [2], yeast  [12], and mammalian cells  [11], establishing a basic model framework for linking gene regulatory mechanisms with distribution data. Building on the telegraph model, transcription models with different gene state-switching mechanisms have been derived, such as those containing multiple gene states, multiple signaling pathways, and positive/negative feedback loops  [13–15].

The telegraph model is widely utilized to fit mRNA expression distributions in single-cell RNA-seq (scRNA-seq) data, owing to its biologically interpretable structure  [11,16–18]. For instance, it accurately models 90% of transcripts in scRNA-seq datasets and outperforms empirical bulk-focused gamma-Poisson models in capturing bimodal distributions  [9]-a hallmark that directly characterizes heterogeneity in isogenic cell populations  [1,12]. The model infers two core parameters: transcriptional burst frequency $b_{f}$ (the rate of switching from inactive to active transcription), and burst size $b_{s}$ (the average mRNA copies produced per active state). Notably, $b_{f}$ and $b_{s}$ estimated by the telegraph model are regarded as effective parameters. Even for genes regulated by more complex regulatory mechanisms, these effective metrics remain widely applicable and can partly reflect genuine changes in transcriptional burst frequency and size  [11,17–20].

Accurate estimation of $b_{f}$ and $b_{s}$ is critical for deciphering the intensity of gene regulation and its contextual dynamics. For example, in mouse embryonic stem (ES) cells, transcription factors *Oct4* and *Nanog* regulate $b_{f}$ post-replication  [21]; in *Escherichia coli* (*E. coli*), over 20 promoters modulate $b_{s}$ in response to growth signals  [8]; and in human T cells, HIV LTR promoters co-regulate both parameters under distinct chromatin environments  [7]. Recent studies have extended the telegraph model from analyzing specific gene circuits to genome-wide transcription distributions in mouse and human cells. These extensions revealed that promoter elements (e.g., TATA box and initiator) synergistically enhance $b_{s}$  [11,22], whereas specific regulators and signaling pathways predominantly target either $b_{f}$ or $b_{s}$  [17,18]. For instance, *MYC*-bound genes exhibit larger $b_{s}$, while *AFF4*-targeted genes show enriched $b_{f}$  [22].

The telegraph model has also gained growing attention for genome-wide identification of DEGs across distinct cell types or states  [9,10,16,19]. This progress relies on efficient algorithms to compute its distribution-for example, the Finite-State Projection (FSP) algorithm  [3,20] and Gauss-Jacobi quadrature method  [9]. Compared to traditional DEGs analysis methods, it offers two key advantages. First, it deciphers DEGs’ intrinsic regulatory changes from a biophysical perspective by linking them to burst frequency $b_{f}$ and size $b_{s}$. For example [19], showed that across mouse CAST and C57 alleles (fibroblasts vs. embryonic stem cells): $b_{f}$ predominantly regulates cell cycle-related DEGs, while $b_{s}$-regulated DEGs enrich in signaling and energy metabolism. Second, it overcomes a key limitation of traditional methods: failure to detect DEGs with unaltered mean but changed expression distributions, making it ideal for capturing heterogeneity-linked differences. For instance [16], found that in mouse brain tissue, the gene *Ndnf* (encoding neuron-derived neurotrophic factor) showed significant differences in inferred burst size between the L6 corticothalamic neuronal subclass and others, despite no detectable change in mRNA mean level.

However, neither the telegraph model nor its extensions address a critical issue: pervasive zero inflation that exists in single-cell transcriptional datasets. Zero inflation stems from two distinct sources: biological zeros (true non-expression, arising from stochastic gene state switching inherent to single-cell heterogeneity) and technical zeros (well-documented artifacts in scRNA-seq, i.e., dropout events like inefficient mRNA capture or amplification failure)  [23,24]. This conflates the zero probability *P*_0_ in mRNA distributions, which incorrectly aggregates biological and technical zeros. Consequently, *P*_0_ is artificially inflated in most mRNA distribution data, severely undermining the telegraph model’s ability to capture the true distributional characteristics  [25]. For instance, the bimodal distribution of paternal *Mbnl2* transcription in mouse fibroblasts is misfitted as unimodal by the telegraph model using standard maximum-likelihood estimation method (MLE)  [11]. In contrast, our newly developed fitting approach-by ensuring precise fitting of *P*_0_ during distribution data fitting-enables the telegraph model to accurately recapitulate bimodal patterns  [26]. Notably, this method also enhances fitting performance for unimodal mRNA distributions in mammalian cells  [3,26].

While zero inflation has been integrated into stochastic gene transcription models, existing approaches primarily incorporate a zero-inflation parameter into the negative binomial distribution  [23,27], a strategy widely adopted for single-cell RNA-seq data analysis  [28–30]. Such models have been demonstrated to identify three classes of DEGs during embryonic development, with each class enriched in distinct biological processes  [23]. However, large-scale single-cell datasets have confirmed that stochastic transcriptional regulation inherently generates bimodal distributions, whereas the negative binomial distribution alone exclusively yields unimodal patterns. Thus, such models generate bimodality solely through technical zero-inflation, and therefore fail to capture the transcriptional burst regulation that underlies bimodality. Therefore, it is imperative to develop a telegraph model integrated with zero inflation to investigate transcriptional regulatory mechanisms and DEGs.

In this study, we developed a zero-inflated telegraph model that integrates technical zero correction with the classical telegraph model’s ability to capture stochastic gene state-switching dynamics. We systematically validated this model using diverse datasets: synthetic data with dropout, extrinsic noise and varied sample sizes; human scRNA-seq data from lupus patient CD14^+^ monocytes  [31] and breast cancer patient CD8^+^ T cells  [32]; and mouse scRNA-seq data from embryonic stem cells  [11,17]. We demonstrated that the model outperforms conventional models in fitting mRNA distributions, reliably estimates transcriptional burst parameters without overfitting, and enhances DEGs detection and their subsequent functional enrichment. Collectively, this model provides a robust, biologically interpretable tool for single-cell transcriptomic analysis.

## Results

### Zero-inflated telegraph model

In the classical telegraph model  [1,3,4,8], as illustrated in the following diagram:


$$\begin{array}{c} \text{gene off}\overset{k_{\text{on}}}{\underset{k_{\text{off}}}{⇄}}\text{gene on}\overset{k_{\text{b}}\;}{\to }\text{mRNA}\overset{k_{\text{d}}\;}{\to }\emptyset \end{array}$$

the gene is proposed to transition stochastically between an active (on) state and an inactive (off) state, governed by first-order rate constants: the activation rate *k*_on_ and inactivation rate *k*_off_. During the active state, mRNA molecules are synthesized at a first-order production rate *k*_b_. For mRNA molecules, their degradation, which includes both decay and dilution during cell division, occurs at a first-order decay rate *k*_d_. Then the burst frequency $b_{f}$ and size $b_{s}$ are expressed as  [1,3]


$$\begin{array}{c} b_{f}=k_{\text{on}},\;b_{s}=\frac{k_{\text{b}}}{k_{\text{off}}}. \end{array}$$

The telegraph model allows for deriving the steady-state exact formulas of mRNA distribution $P_{m}$ through solving master equations  [3,15]:


$$\begin{array}{c} P_{m}=\frac{1}{m!}\frac{d^{m}F(z)}{dz^{m}}{|}_{z=0},\;F(z)=\sum_{m=0}^{\infty }P_{m}z^{m}={\;}_{1}F_{1}(k_{\text{on}}/k_{\text{d}};k_{\text{on}}/k_{\text{d}}+k_{\text{off}}/k_{\text{d}};k_{\text{b}}(z-1)/k_{\text{d}}), \end{array}$$

where ${\;}_{1}F_{1}$ denotes the confluent hypergeometric function  [33]. Note that the exact formula of $P_{m}$ is expressed in terms of the ratios $k_{\text{on}}/k_{\text{d}},k_{\text{off}}/k_{\text{d}}$ and $k_{\text{b}}/k_{\text{d}}$. To improve clarity, we standardize the degradation rate *k*_d_ by setting *k*_d_ = 1  [1,20]. Accordingly, all model parameters should be interpreted as the ratio of their actual values to *k*_d_. When applying the telegraph model to the fitting of genome-wide distribution data, directly employing its exact expression for computations yields unsatisfactory precision and efficiency  [3,10]. Instead, we can treat $P_{m}$ of the telegraph model as a beta-Poisson distribution  [4,9]


$$\begin{array}{c} P_{m}=\frac{\Gamma (k_{\text{on}}+k_{\text{off}})}{\Gamma (k_{\text{on}})\Gamma (k_{\text{off}})}\frac{k_{\text{b}}^{m}}{m!}\int_{0}^{1}e^{-k_{\text{b}}s}s^{k_{\text{on}}+m-1}(1-s{)}^{k_{\text{off}}-1}ds, \end{array}$$ (1)

which can be efficiently computed via the Gauss-Jacobi quadrature method  [9].

The telegraph model has a well-known special form corresponding to the negative binomial distribution. This limiting case arises when the inactivation rate *k*_off_ and mRNA synthesis rate *k*_b_ are both sufficiently large, while the burst frequency *k*_on_ and size $b_{s}=k_{\text{b}}/k_{\text{off}}$ stay within regular ranges. Under this scenario, genes remain predominantly in the inactive state; upon activation, they rapidly produce abundant transcripts, and the resulting expression counts follow a negative binomial distribution  [3]:


$$\begin{array}{c} P_{m}=(\frac{1}{1+b_{s}}{)}^{b_{f}}\frac{\Gamma (b_{f}+m)}{\Gamma (b_{f})\Gamma (1+m)}(1-\frac{1}{1+b_{s}}{)}^{m}. \end{array}$$ (2)

It has been shown that a substantial portion of genome-wide distribution data in mouse and human cells can be described by the negative binomial distribution  [11,19]. However, it can be mathematically proven that this distribution only generates unimodal distributions, and thus fails to capture the bimodal distributions that characterize heterogeneity  [1,2,11].

To use either the telegraph model or negative binomial distribution to describe read counts while accounting for excess zero inflation, we introduce the average technical zero rate θ for each cell, where technical zero refers to zeros caused by technical failures in mRNA detection. Let *N* denote the total number of cells in an isogenic cell population, and $N_{m}(m=0,1,2,\cdots )$ denote the number of cells generating exactly *m* mRNA copies (true mRNA counts). Let ${\bar{P}}_{m}$ represent the observed distribution that incorporates these technical zeros, while $P_{m}=N_{m}/N$ denotes the true distribution generated by inherent gene regulatory mechanisms. Then we have


$$\begin{array}{c} {\bar{P}}_{0}=\frac{N_{0}+\theta (N-N_{0})}{N}=P_{0}+\theta (1-P_{0}),\;{\bar{P}}_{m}=\frac{N_{m}-\theta N_{m}}{N}=(1-\theta )P_{m},\;m\geq 1. \end{array}$$ (3)

When $P_{m}$ is expressed via Eq. (1), ${\bar{P}}_{m}$ denotes the exact mRNA distribution formula for the zero-inflated telegraph model; when expressed via Eq. (2), this formulation corresponds to the zero-inflated negative binomial (ZINB) model  [23], which is widely adopted in modern single-cell transcriptomic analyses  [28–30]. Notably, although both the zero-inflated telegraph and ZINB models can produce bimodal mRNA distributions, their underlying mechanisms differ substantially. The telegraph model intrinsically generates bimodal patterns from random transitions between the gene’s active and inactive states during transcriptional bursting. By contrast, ZINB achieves bimodality by combining the non-zero peak derived from the negative binomial distribution and an extra zero peak introduced by the technical zero rate θ. We proved that Eq. (3) ensures a unique parameter set $\left(k_{\text{on}},k_{\text{off}},k_{\text{b}},\theta \right)$ exists for every observed distribution ${\bar{P}}_{m},m=0,1,\cdots$ (Methods).

### Zero-inflated telegraph model robustly captures mRNA distribution with dropout

We first examined differences among mRNA distributions generated by the telegraph model, ZINB model, and zero-inflated telegraph model. It has been established that the telegraph model can generate only three distribution types: a bimodal distribution with both zero and non-zero peaks, and unimodal distributions with either a sole zero peak or a sole non-zero peak  [4]. We generated synthetic datasets containing 4,000 cells using the stochastic simulation algorithm (SSA) under the zero-inflated telegraph model with a technical zero rate θ=0.1. We then fitted these synthetic datasets to the telegraph and ZINB models separately using MLE to test their fitting performance; see Fig 1A. When the underlying core telegraph process yields either a bimodal or a non-zero-peak unimodal distribution, the zero-inflated telegraph model can output bimodal data. While the ZINB model can visually produce bimodal curves when fitted to such data, the valley dividing the zero peak and the secondary peak is fixed rigidly at mRNA count *m* = 1. This artifact causes misalignment in both the inter-peak valley shape and the location of the secondary peak. By contrast, the telegraph model may misclassify these bimodal datasets as unimodal. For parameters where the core telegraph process generates zero-peak unimodal distributions, the zero-inflated telegraph model preserves this unimodal signature. Yet both the telegraph and ZINB models fit such data poorly; notably, the ZINB model may yield bimodal profiles instead of the true unimodal shape.

**Fig 1:**
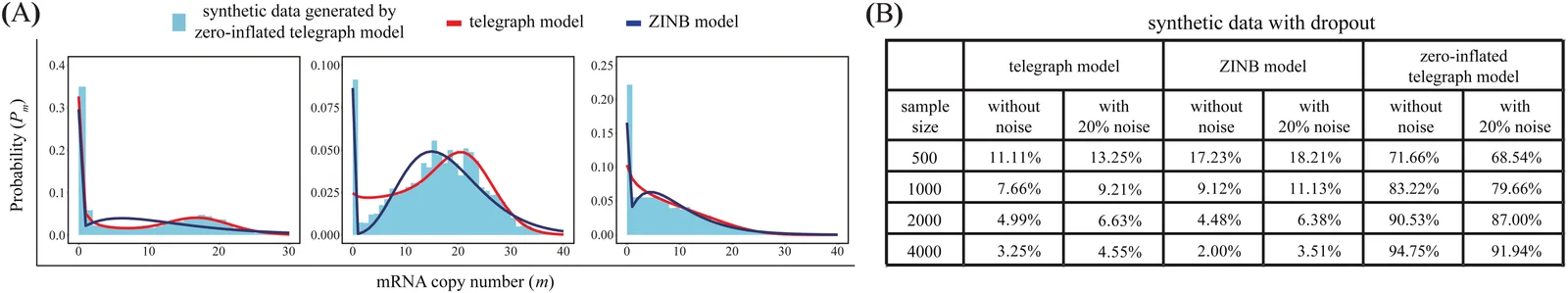
Quantitative differences in mRNA distributions among the telegraph model, ZINB model and zero-inflated telegraph model. **(A)** Fitting performance of the telegraph and ZINB models against mRNA distribution data simulated from the zero-inflated telegraph model. All synthetic datasets are generated with 4000 cell samples and fixed *k*_b_ = 30 and θ=0.1. Three distinct parameter regimes are displayed: left panel $k_{\text{on}}=k_{\text{off}}=0.2$, middle panel $k_{\text{on}}=2,k_{\text{off}}=1$ and right panel $k_{\text{on}}=1,k_{\text{off}}=2$. **(B)** Synthetic distribution data (with dropout events) were generated using the telegraph model across sample sizes (*N* = 500, 1000, 2000, 4000) and with/without 20% extrinsic noise. All parameters satisfied $k_{\text{on}},k_{\text{off}}\in (0,3)$ and $k_{\text{b}}\in (10,30)$. The zero-inflated telegraph model maintains correct selection rates of nearly 70% or higher, outperforming the other two models.

We further examined whether the distributions generated by the three models are distinguishable via model fitting and selection. To this end, we randomly sampled 10^4^ parameter sets for the telegraph model, with $k_{\text{on}},k_{\text{off}}\in (0,3),k_{\text{b}}\in (10,30)$. We then employed the SSA to generate synthetic distribution data for each parameter set across varying cell sample sizes (*N* = 500, 1000, 2000, 4000). Next, we introduced the dropout model  [23,35] to add dropout events to the synthetic distribution data (S1 Appendix). Each of the three models then fits these dropout-containing synthetic datasets via MLE, with the corrected Akaike information criterion (AICc) subsequently applied to identify the optimal model for describing the data (S1 Appendix). AICc imposes a penalty based on parameter count: 4 for the zero-inflated telegraph model and 3 for the other two. As illustrated in Fig 1B, over 70% of the synthetic datasets were correctly identified as best described by the zero-inflated telegraph model. This indicates that the other two models’ distributions fail to accurately capture the synthetic profiles, and their pronounced deviations cannot be masked by the AICc penalty from the zero-inflated telegraph model’s additional parameter.

To assess the robustness of this finding, we further introduced extrinsic noise into the telegraph model’s parameters $k_{\text{on}},k_{\text{off}},k_{\text{b}}$: each parameter was redefined as a log-normally distributed random variable, retaining its original mean while setting the standard deviation to 20% of the mean (i.e., a 20% noise level)  [34,36]. Synthetic distribution data with extrinsic noise were first generated using SSA; dropout events were then added to these data following the same procedure as above. Notably, while the selection rate of the zero-inflated telegraph model decreased slightly compared to the noise-free scenario, it remained above 70% across most sample sizes. Even for the smallest sample size *N* = 500, the rate was only marginally lower at 68.5%; see Fig 1B. Collectively, these results highlight the zero-inflated telegraph model’s robust reliability in describing complex mRNA distributions with technical artifacts (e.g., dropout) and biological noise. In contrast, the other two models lack sufficient accuracy to match the target distributions.

### Zero-inflated telegraph model reliably estimates effective transcriptional burst frequency and size

We first evaluated how well the telegraph model, ZINB model, and zero-inflated telegraph model estimate burst frequency $b_{f}$ and burst size $b_{s}$, using the 10^4^ groups of dropout-containing synthetic distributions described in Fig 1B. For each model, we plotted 10^4^ points in Fig 2A, where each point compares the estimated $b_{f}$ (or $b_{s}$) against its true value. Ideally, if all points cluster tightly along the 1:1 reference line, it indicates consistent estimates of effective burst parameters (with a Pearson correlation coefficient, PCC = 1). In contrast, points straying far from this line signal unreliable estimation, typically accompanied by an extremely low PCC.

**Fig 2:**
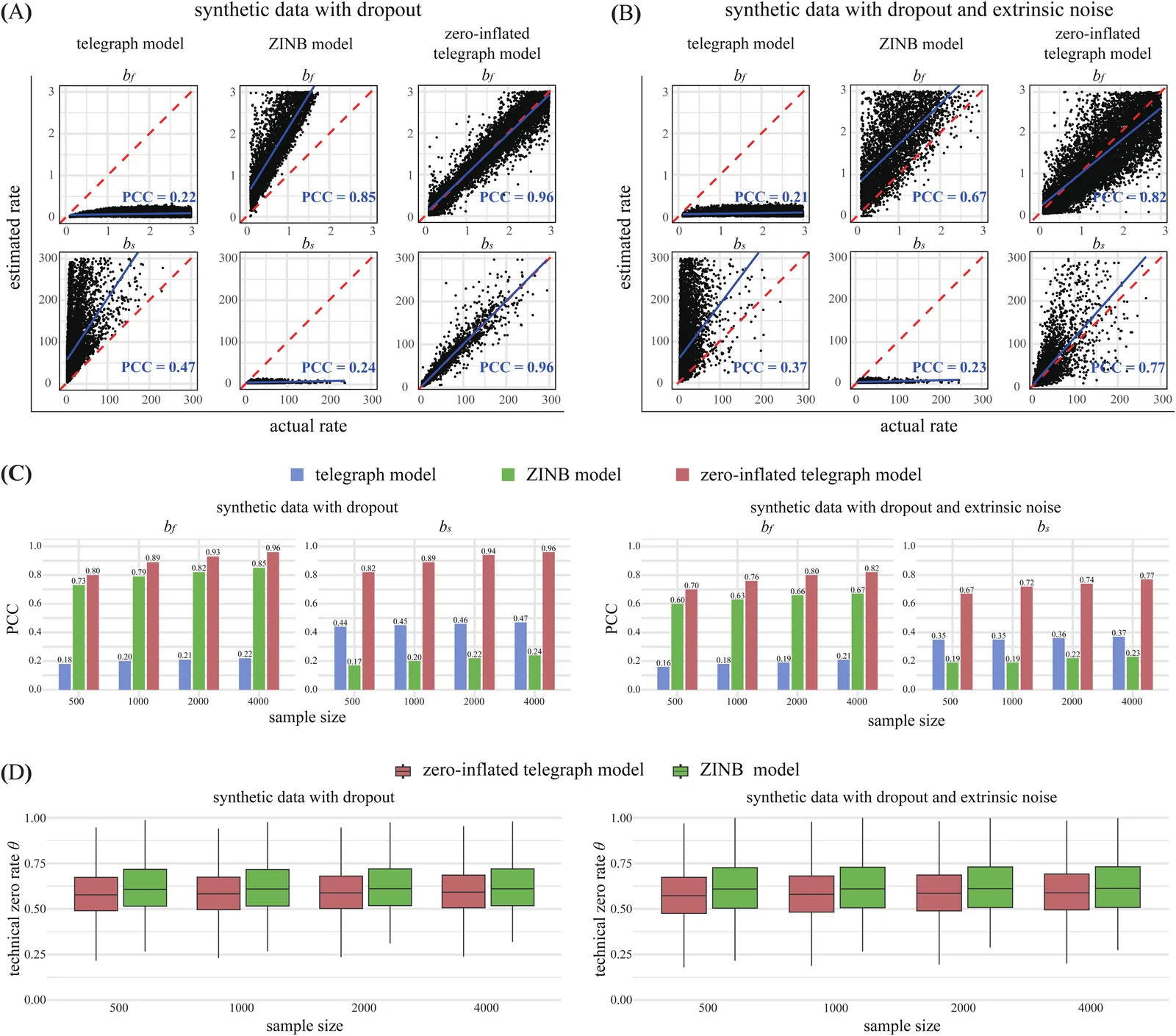
Reliable transcriptional burst parameter inference of the zero-inflated telegraph model. Synthetic distribution data (with dropout events) were generated via the telegraph model across sample sizes (*N* = 500, 1000, 2000, 4000) and with/without 20% extrinsic noise. All parameters were constrained to $k_{\text{on}},k_{\text{off}}\in (0,3)$ and $k_{\text{b}}\in (10,30)$. **(A)** Estimation of $b_{f}$ and $b_{s}$ (no extrinsic noise, *N* = 4000): Zero-inflated telegraph model estimates cluster along the 1:1 line (PCC = 0.96); telegraph model underestimates $b_{f}$ and overestimates $b_{s}$, while ZINB model shows opposite bias. **(B)** Estimation with 20% extrinsic noise: All models have reduced precision, but zero-inflated telegraph model remains robust (PCC > 0.75) with consistent bias patterns of the three models. **(C)** Estimation across different sample sizes (with/without noise): Zero-inflated telegraph model outperforms other models (PCC > 0.7, except 0.67 at *N* = 500 with 20% extrinsic noise); telegraph model and ZINB model struggles with inferring $b_{f}$ and $b_{s}$, respectively. **(D)** θ distribution of two zero-inflated models: θ is similar and stable across sample sizes and extrinsic noise, confirming robust dropout rate inference.

At the large sample size *N* = 4000 (Fig 2A), the $b_{f}$ and $b_{s}$ estimates from the telegraph model departed markedly from the 1:1 line (PCC < 0.5): $b_{f}$ points consistently lay below the line (indicating underestimation), while $b_{s}$ points hovered above it (indicating overestimation). The ZINB model showed the opposite bias: overestimating $b_{f}$ and underestimating $b_{s}$. In striking contrast, the zero-inflated telegraph model’s estimates for both $b_{f}$ and $b_{s}$ clustered tightly along the 1:1 line, with high PCC of 0.96. This underscores the model’s ability to reliably estimate burst parameters. These trends persisted when fitting dropout-containing synthetic data with 20% extrinsic noise added to each system parameter (Fig 2B). Notably, estimation accuracy degraded across all models relative to the noise-free scenario, yet their patterns of overestimation or underestimation for $b_{f}$ and $b_{s}$ remained unchanged. Crucially, the zero-inflated telegraph model maintained its advantage on inferring $b_{f}$ and $b_{s}$: despite the noise-induced decline in precision, it retained robust performance with a PCC above 0.75, reinforcing its superiority in reliably estimating burst parameters even under noisy conditions.

We further compared the estimation of $b_{f}$ and $b_{s}$ across the three models under varying sample sizes *N* = 500, 1000, 2000, 4000; see Fig 2C and Fig A in S1 Appendix. Several consistent principles emerge. First, the zero-inflated telegraph model consistently outperforms the others, reliably estimating both $b_{f}$ and $b_{s}$ with PCC > 0.7 across almost all sample sizes, whether with or without extrinsic noise. The only exception is a modest PCC of 0.67 under the smallest sample size *N* = 500 with extrinsic noise. Second, the telegraph model struggles more with $b_{f}$ (PCC < 0.25) than with $b_{s}$, while the ZINB model shows the reverse: it fares better with $b_{f}$ but poorly with $b_{s}$ (PCC < 0.25). This means neither model can simultaneously estimate both $b_{f}$ and $b_{s}$ reliably. Taken together, these findings highlight the zero-inflated telegraph model’s unique robustness: it maintains reliable parameter estimation across diverse sample sizes and noise conditions, whereas the other two models are inherently limited by their inability to balance accurate estimation of both burst frequency and size.

We finally examined the estimation of the technical zero rate θ for the ZINB model and zero-inflated telegraph model. Notably, there is no “true” θ value, as dropout-containing synthetic distributions were generated by randomly introducing dropout events for each gene and cell (S1 Appendix). Fig 2D shows θ estimates for both zero-inflated models, with and without extrinsic noise. The two models yielded consistently similar θ values, with the ZINB model estimating slightly higher values. This indicates that the zero-inflated telegraph model’s superior performance in describing synthetic data does not stem from better capturing dropout strength, but rather from the telegraph model’s greater power to capture inherent gene regulatory patterns. Equally striking, θ estimates remained stable across varying sample sizes and regardless of extrinsic noise. This underscores the robustness of zero-inflated models in estimating the dropout rate even when experimental conditions change. We also explore the correlation between the estimated θ and the estimation reliability of $b_{f}$ and $b_{s}$. Using the 10^4^ synthetic datasets with dropout (sample sizes *N* = 500, 1000, 2000, 4000, see Fig 1B), we calculated the absolute relative errors of $b_{f}$ and $b_{s}$. The results reveal a weak positive correlation between these errors and inferred θ, with PCC ranging between 0.1 and 0.4 (Fig  B in S1 Appendix).

### Zero-inflated telegraph model exhibits no over-fitting

We investigated how the zero-inflated telegraph model estimates burst parameters when fitting synthetic data from simpler models, focusing on sample sizes *N* = 500, 1000, 2000, 4000. Using SSA, we generated 10^4^ synthetic distributions from the telegraph model with parameters $k_{\text{on}},k_{\text{off}}\in (0,3)$ and $k_{\text{b}}\in (10,30)$, alongside 10^4^ dropout-containing distributions from the ZINB model. Notably, both sets share same burst frequency $b_{f}$and burst size $b_{s}$.

For telegraph model-generated data, we fitted three models and plotted $b_{f}$ and $b_{s}$ estimates against their true values Fig 3A and Fig C in S1 Appendix: the telegraph model itself yielded accurate estimates with a PCC near 1. In contrast, the ZINB model’s estimates strayed from the 1:1 line, with overestimated $b_{f}$ and underestimated $b_{s}$, consistent with Fig 2A. Critically, the zero-inflated telegraph model’s estimates clustered tightly along the 1:1 line (PCC approaching 1). Its estimated technical zero rate θ was tiny across all sample sizes (θ<0.015) and smaller than that from the ZINB model (Fig 3B), highlighting its superior ability to distinguish dropout events. For dropout-containing data from the ZINB model, the telegraph model underestimated $b_{f}$ and overestimated $b_{s}$, while both zero-inflated models produced similarly reliable burst parameter estimates Fig 3C and Fig C in S1 Appendix. Their θ estimates also aligned (Fig 3D), showing that the zero-inflated telegraph model captures dropout rates well even when fitting simpler data. Together, these findings confirm the zero-inflated telegraph model’s robust ability to balance flexibility to capture dropouts and parsimony to avoid overparameterization.

**Fig 3:**
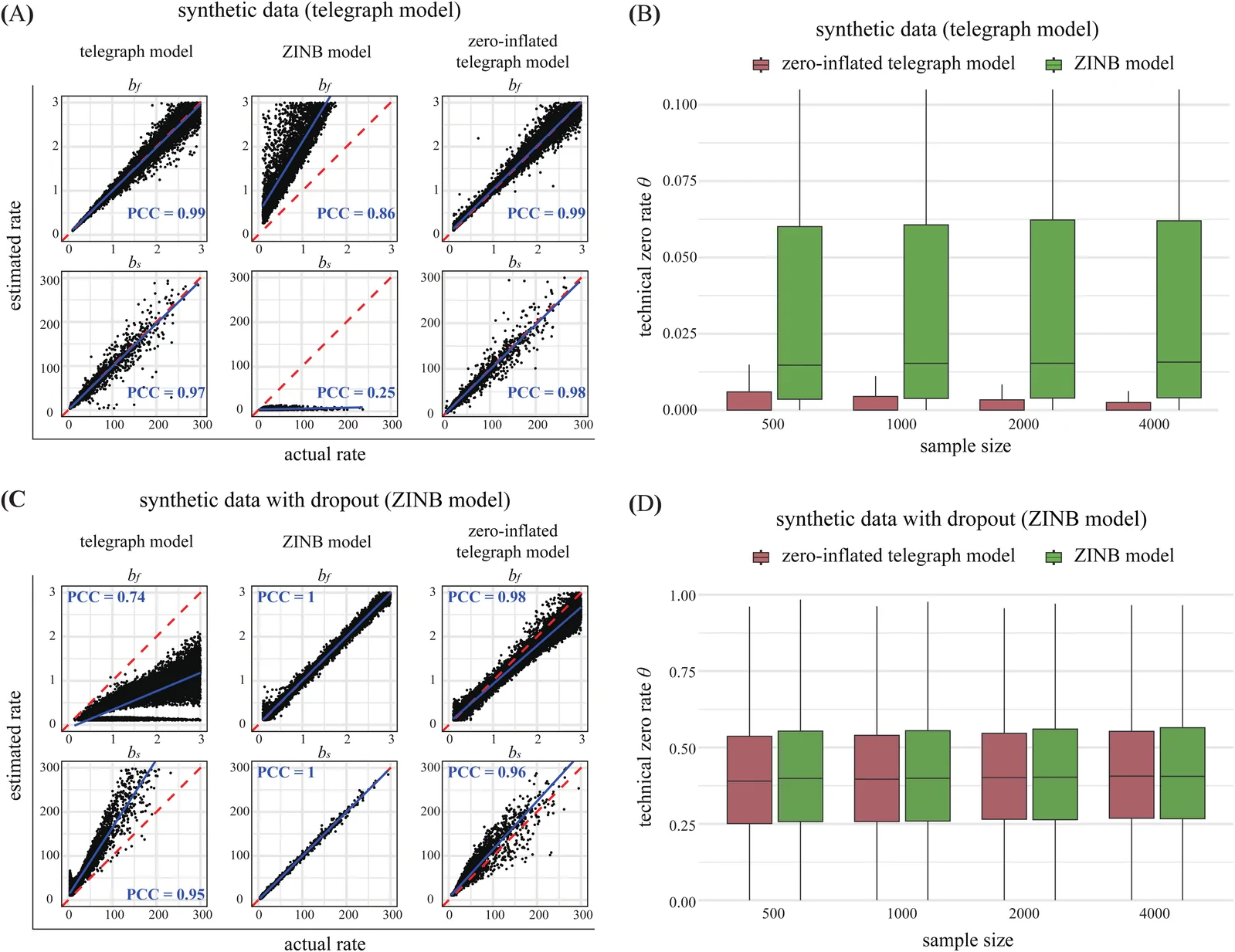
Zero-inflated telegraph model fitting simpler synthetic data. **(A)** For telegraph model-generated data (*N* = 4000), the zero-inflated telegraph model’s $b_{f}$ and $b_{s}$ estimates cluster tightly along the 1:1 reference line (PCC approaching 1), while the ZINB model shows obvious $b_{f}$ and $b_{s}$ estimation bias. **(B)** For telegraph model-generated data, the zero-inflated telegraph model has tiny θ (θ<0.015) across sample sizes, smaller than those of the ZINB model. **(C)** For ZINB model-generated dropout-containing data (*N* = 4000), the telegraph model underestimates $b_{f}$ and overestimates $b_{s}$, while both zero-inflated models give reliable $b_{f}$ and $b_{s}$ estimates. **(D)** For ZINB model-generated dropout-containing data, the two zero-inflated models have aligned θ estimates, confirming the zero-inflated telegraph model’s ability to capture dropout rates. For both the telegraph model and ZINB model, all parameters were constrained to $k_{\text{on}},k_{\text{off}}\in (0,3)$ and $k_{\text{b}}\in (10,30)$, with $b_{f}=k_{\text{on}}$ and $b_{s}=k_{\text{b}}/k_{\text{off}}$.

### Generalization evaluation across different gene regulatory models

In previous analyses, synthetic datasets were generated based on the basic telegraph model and negative binomial distribution. Inspired by general approach of generating synthetic data based on complex gene regulatory networks for algorithm validation  [37], we further tested the generalization capacity of our zero-inflated telegraph model using the three-state model, as shown in the following diagram [38]:


$$\begin{array}{c} off\overset{k_{\text{on,1}}\;}{\to }off\;1\overset{k_{\text{on,2}}\;}{\to }on,\;on\overset{k_{off}\;}{\to }off,\;on\overset{k_{b}\;}{\to }on+mRNA,\;mRNA\overset{k_{d}\;}{\to }\emptyset . \end{array}$$

The three-state model is a biologically motivated extension of the classical telegraph model. To fit experimental observations, it expands the single inactive state into two sequential states controlled by separate activation rates *k*_on,1_ and *k*_on,2_. This modification resolves the telegraph model’s drawback of assuming exponentially distributed inactive durations, which fails to explain the non-exponential peak patterns widely detected across prokaryotic and eukaryotic genes  [39,40]. Mechanistically, the three-state structure mimics multi-step promoter events such as chromatin remodeling, and also reproduces the refractory state of transcription  [15].

All synthetic datasets were generated using SSA with 4000 cells. We created 10^4^ datasets for the three-state model with parameters sampled within fixed intervals: $k_{\text{on,1}}=k_{\text{on,2}}\in (0,6),k_{\text{off}}\in (0,3)$ and $k_{b}\in (10,30)$. We set $k_{\text{on,1}}=k_{\text{on,2}}$ to highlight the multi-step activation mechanism that distinguishes the three-state model from the telegraph model. Specifically, the three-state model degenerates into the telegraph model when $k_{\text{on,1}}\gg k_{\text{on,2}}$. Meanwhile, prior evidence confirms that equal values of *k*_on,1_ and *k*_on,2_ yield the maximum deviation of transcriptional noise from the telegraph model  [15]. For the three-state model, we prepared two types of synthetic data: raw datasets free of technical zero inflation, and datasets with dropout events to simulate sequencing-derived biases. Next, we benchmarked the performance of the classical telegraph model, ZINB model, and our newly proposed zero-inflated telegraph model.

Three major conclusions were drawn from the comparative analyses. First, we adopted the Hellinger distance (HD) to quantify distribution fitting performance (Fig 4A). On datasets free of technical zeros, the zero-inflated telegraph model and the classical telegraph model achieved the lowest HD values and optimal fitting results, whereas the ZINB model performed the worst. When technical zero inflation was introduced, the zero-inflated telegraph model still maintained the minimal HD, followed by the ZINB model. This performance difference demonstrates that integrating a dedicated zero-inflation correction is indispensable for analyzing scRNA-seq data contaminated by dropout artifacts.

**Fig 4:**
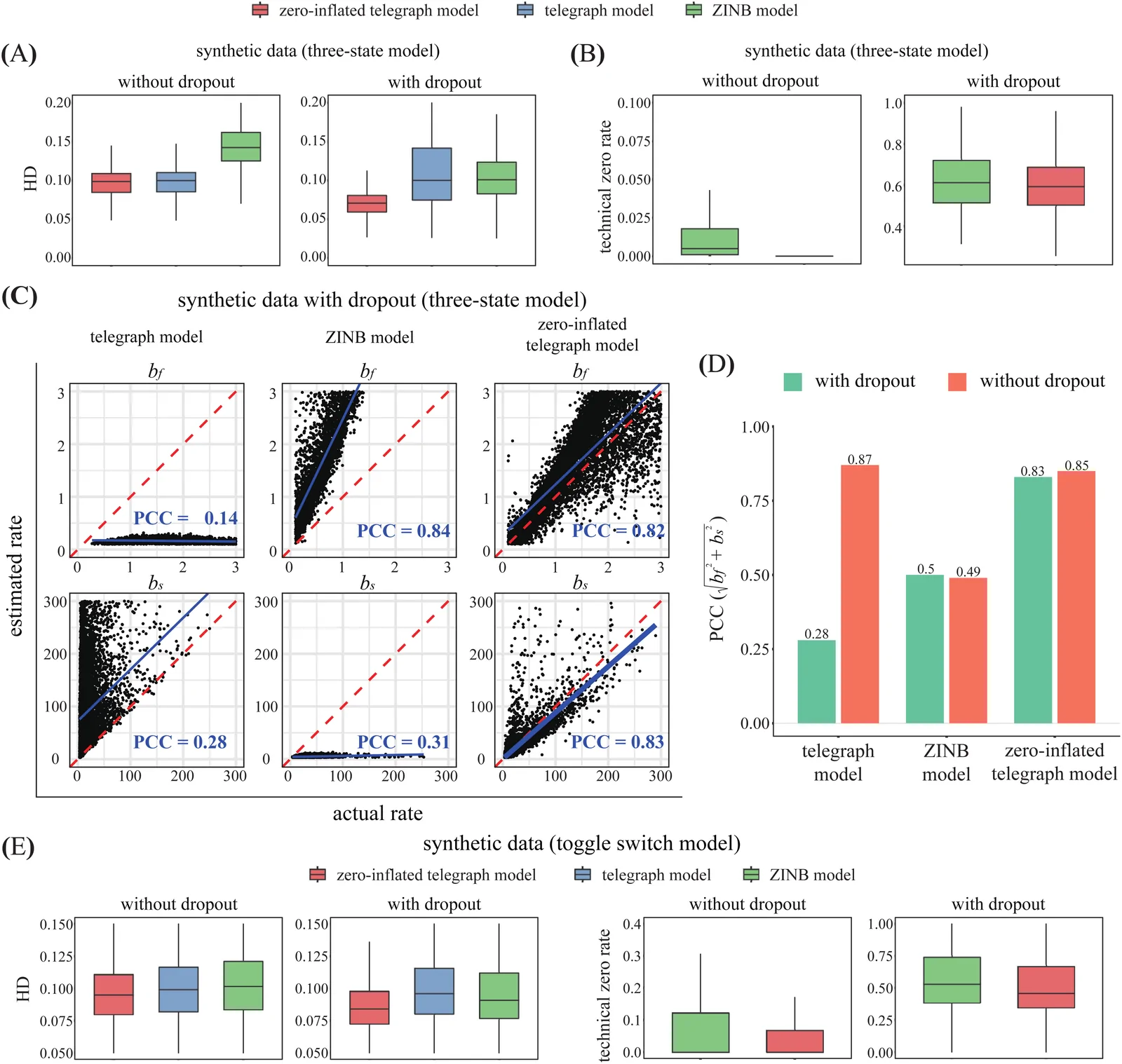
Evaluation of model generalization using synthetic data from the three-state transcription model. Data were generated with parameters constrained to $k_{\text{on,1}}=k_{\text{on,2}}\in (0,6),k_{\text{off}}\in (0,3)$ and $k_{\text{b}}\in (10,30)$. **(A)** Hellinger distance (HD) results show that the zero-inflated telegraph model outperforms the classic telegraph model and ZINB model in distribution fitting. **(B)** Estimates of the technical zero rate θ confirm that the zero-inflated telegraph model accurately distinguishes biological zeros from technical artifacts. **(C)** The zero-inflated telegraph model exhibits relatively higher accuracy for inferring burst frequency $b_{f}$ and burst size $b_{s}$ of the three-state model. **(D)** The zero-inflated telegraph model effectively tracks relative changes in overall transcriptional kinetics of the three-state regulatory system, whereas the telegraph model and ZINB model fail to do so. **(E)** Comparative fitting performance on synthetic genetic toggle switch datasets. In the absence of technical dropout noise, the zero-inflated telegraph model yields lower HD and smaller estimated technical zero rate θ than the ZINB model; with dropout noise introduced, it retains minimal HD while generating broadly consistent θ estimates relative to ZINB.

Second, we evaluated the estimation of the technical zero rate θ (Fig 4B). For synthetic data without technical zeros, our model yielded extremely small θ values, which were lower than those estimated by the ZINB model. In contrast, the two zero-inflated models produced comparable θ values when fitting datasets with dropout events. This result confirms that our model can accurately distinguish biological zeros from sequencing-induced technical zeros, and reliably judge whether input data contains zero-inflation artifacts.

Third, we assessed the inference accuracy of burst frequency $b_{f}$ and burst size $b_{s}$. On datasets without zero inflation, the zero-inflated telegraph model showed comparable accuracy to the telegraph model, and both performed considerably better than the ZINB model (Fig  D in S1 Appendix). This indicates that the negative binomial distribution in ZINB is unable to capture the mechanisms of the three-state gene regulatory model. When zero inflation was introduced, the zero-inflated telegraph model still achieved satisfactory parameter estimation, with PCC values above 0.8 for both $b_{f}$ and $b_{s}$. Its performance was far better than that of the telegraph model and ZINB (Fig 4C), demonstrating that modeling zero inflation and intrinsic regulatory mechanisms is essential for accurate parameter inference.

Though our model inevitably produces quantitative deviations when fitting data generated from more complex three-state model, we mainly examined whether its estimates reflect true parameter changes. We used the root sum of squares (RSS), defined as $\sqrt{b_{f}^{2}+b_{s}^{2}}$, to characterize overall parameter variations. The RSS estimated by our model was strongly positively correlated with the true values, while neither the telegraph model nor ZINB model could reproduce such trends (Fig 4D). These results verify that our model reliably captures relative changes in transcriptional kinetics for the three-state gene regulatory system.

To further examine the generalization capacity of the zero-inflated telegraph model across distinct gene regulatory architectures, we generated synthetic datasets using the canonical genetic toggle switch circuit  [37], which describes a two-gene system where each gene product represses the transcription of its counterpart. We randomly sampled 5,000 parameter groups with repression rates confined to (0,3) and gene product synthesis rates within (10,30), while fixing the degradation rate at unity. Synthetic single-cell expression profiles containing 4,000 cells per condition were simulated via the Python package gillespy2, yielding a total of 10^4^ synthetic distribution datasets of two genes. We then fitted all synthetic data using three competing frameworks: the telegraph model, ZINB model, and our zero-inflated telegraph model, with quantitative comparisons summarized in Fig 4E.

For synthetic datasets free of sequencing dropout artifacts, the zero-inflated telegraph model and telegraph model yielded comparable HD values, both lower than those obtained from the ZINB model. Meanwhile, the technical zero rate θ estimated by our model remained smaller than that of the ZINB model. When sequencing dropout noise was incorporated into simulated profiles, the zero-inflated telegraph model produced the lowest HD, with its inferred θ values comparable with those output by the ZINB model. Collectively, these observations indicate that the zero-inflated telegraph model maintains robust performance in distribution fitting and avoids overfitting risks, even when applied to expression data generated from separate regulatory architectures.

### Zero-inflated telegraph model exhibits superior performance in DEGs analysis

We investigated the performance of three models-the telegraph model, ZINB model, and zero-inflated telegraph model-in identifying DEGs from single-cell mRNA distribution data. For this analysis, we utilized distribution datasets derived from a scRNA-seq experiment of peripheral blood mononuclear cells isolated from lupus patients treated with interferon-beta (IFN-β)  [31]. Specifically, we focused on CD14^+^ monocytes (a key myeloid subset responsive to IFN-β) and analyzed 15,701 genes retained after quality control. From these 15,701 genes, we randomly selected 10,000 genes for subsequent model fitting and DEGs analysis.

We fitted the zero-inflated telegraph model for the 10,000 randomly selected genes to estimate three parameters $\left(k_{\text{on}},k_{\text{off}},k_{\text{b}}\right)$. These parameter estimates served as the control group. To simulate DEGs, we first randomly selected 2,000 parameter sets from the 10,000 parameter estimates of the control group, and artificially perturbed one parameter by 2-fold in each selected set. These 2,000 perturbed parameter sets, together with the remaining 8,000 unchanged parameter sets from the control group, constituted the 10,000 parameter sets for the DEGs group. Using the telegraph model, we then generated dropout-containing synthetic distributions for both control and DEGs groups under four cell sample sizes: *N* = 100, 500, 1000, 2000. We applied the three models to the synthetic datasets to distinguish DEGs from non-DEGs, with implementation details following pipelines adapted from prior protocols  [9,23]. Details of the DEGs detection pipeline are in the S1 Appendix. For performance quantification, we constructed Receiver Operating Characteristic (ROC) curves and computed the Area Under the ROC Curve (AUC) for each model at all four sample sizes: AUC = 1 indicates perfect DEGs detection, while AUC = 0.5 indicates random performance.

As shown in Fig 5, the zero-inflated telegraph model achieved the highest AUC across all sample sizes, followed by the ZINB model, with the telegraph model exhibiting the lowest performance. This result underscores two considerations for DEG analysis: (1) Accounting for zero inflation is essential to avoid misclassifying non-DEGs that arise from spurious zeros, and (2) incorporating the telegraph-like on-off switching dynamics improves detection of DEGs driven by changes in gene regulation kinetics. Notably, the AUC values of the two zero-inflated models converged at larger sample sizes. However, at the smallest sample size *N* = 100, the zero-inflated telegraph model outperformed the ZINB model by a noticeable AUC margin. This observation aligns with prior findings that gene expression in mammalian cells is inherently bursty  [3,7], and further demonstrates that the zero-inflated telegraph model enables more accurate identification of DEGs, particularly in contexts with limited sample sizes.

**Fig 5:**
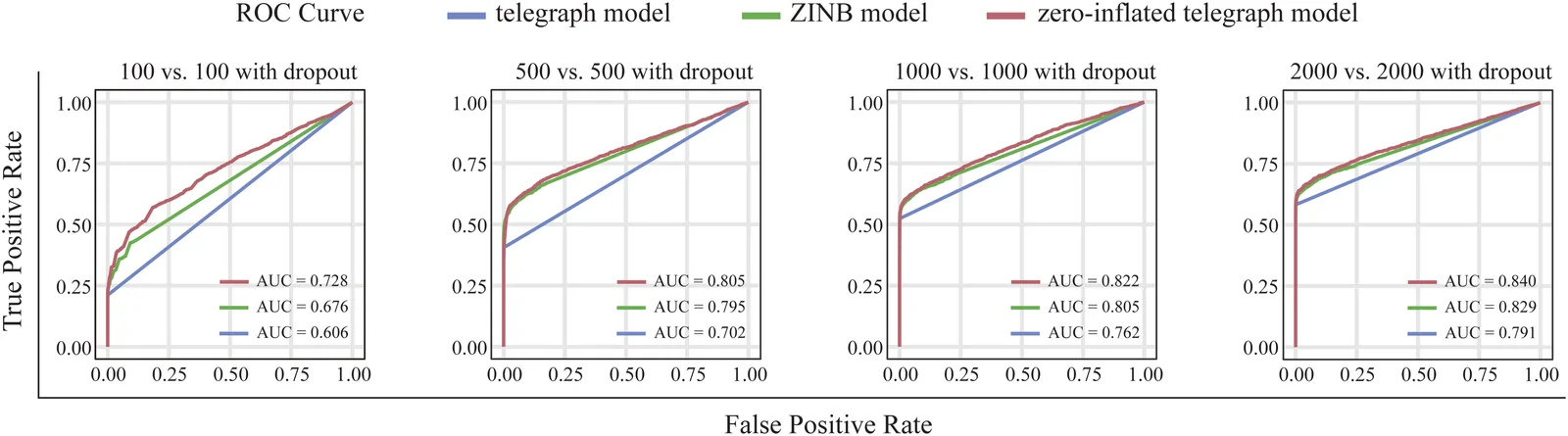
DEGs detection performance of different models. Synthetic datasets simulating DEGs (from telegraph model-generated dropout-containing data, *N* = 100, 500, 1000, 2000) were used to test three models. The zero-inflated telegraph model achieved the highest AUC across all sample sizes (notably superior at small ***N*)**, followed by the ZINB model, with the telegraph model performing lowest.

### Zero-inflated telegraph model enhances DEGs detection in human scRNA-seq data

We validated the zero-inflated telegraph model using two distinct datasets, each composed of two comparative subgroups, followed by model fitting, parameter estimation, and DEGs analysis to verify the model’s performance. The first dataset focuses on CD14^+^ monocytes isolated from peripheral blood mononuclear cells (PBMCs) of systemic lupus erythematosus (SLE) patients  [31]. It comprises two groups: 2,931 cells from the unstimulated control group and 2,765 cells from the IFN-β treated group, respectively, with 15,701 genes profiled across all cells. These cells were derived from multiplexed droplet scRNA-seq experiments, which supports investigating cell-type-specific transcriptional responses to IFN-β stimulation. The second dataset centers on tumor-infiltrating lymphocytes (TILs) isolated from patients with triple-negative breast cancer (TNBC)  [32]. For comparative analysis, two CD8^+^ T cell clusters were selected: CD8^+^ tissue-resident memory T (CD8^+^T${\;}_{rm}$) cells and CD8^+^ non-tissue-resident memory (CD8^+^non-T${\;}_{rm}$) cells, comprising 606 and 1,097 cells, respectively, with 10,061 genes analyzed. These subsets were initially identified through scRNA-seq of TILs, and their transcriptional heterogeneity is crucial for elucidating the functional differences and prognostic significance of CD8^+^ T cell populations in TNBC.

We applied three models to fit the distribution data from 4 groups (derived from PBMCs and TILs) and computed HD to quantify the fitting performance. As shown in Fig 6A, the ZINB model yields significantly lower HD than the telegraph model, suggesting that capturing zero inflation may be more critical than accounting for the inherent gene state-switching mechanism when fitting genome-wide scRNA-seq transcriptomic data in human cells. Furthermore, the zero-inflated telegraph model outperforms the ZINB model with even lower HD values, indicating that incorporating detailed gene regulatory mechanisms also plays a pivotal role in accurate data fitting. To further dissect the balance between capturing zero inflation and gene state switching, we plotted the estimated technical zero rate θ against mean expression levels for both PBMCs and TILs datasets (Fig 6B). The results reveal that θ decreases with increasing mRNA mean, implying that dropout events are rare for observed highly expressed genes, and thus these genes may be better described by the telegraph model than the ZINB model. Collectively, our findings show that capturing zero inflation is more critical for fitting scRNA-seq distribution data, while accounting for inherent gene regulatory mechanisms further improves it. By integrating both, the zero-inflated telegraph model offers superior fitting for genome-wide scRNA-seq data.

**Fig 6:**
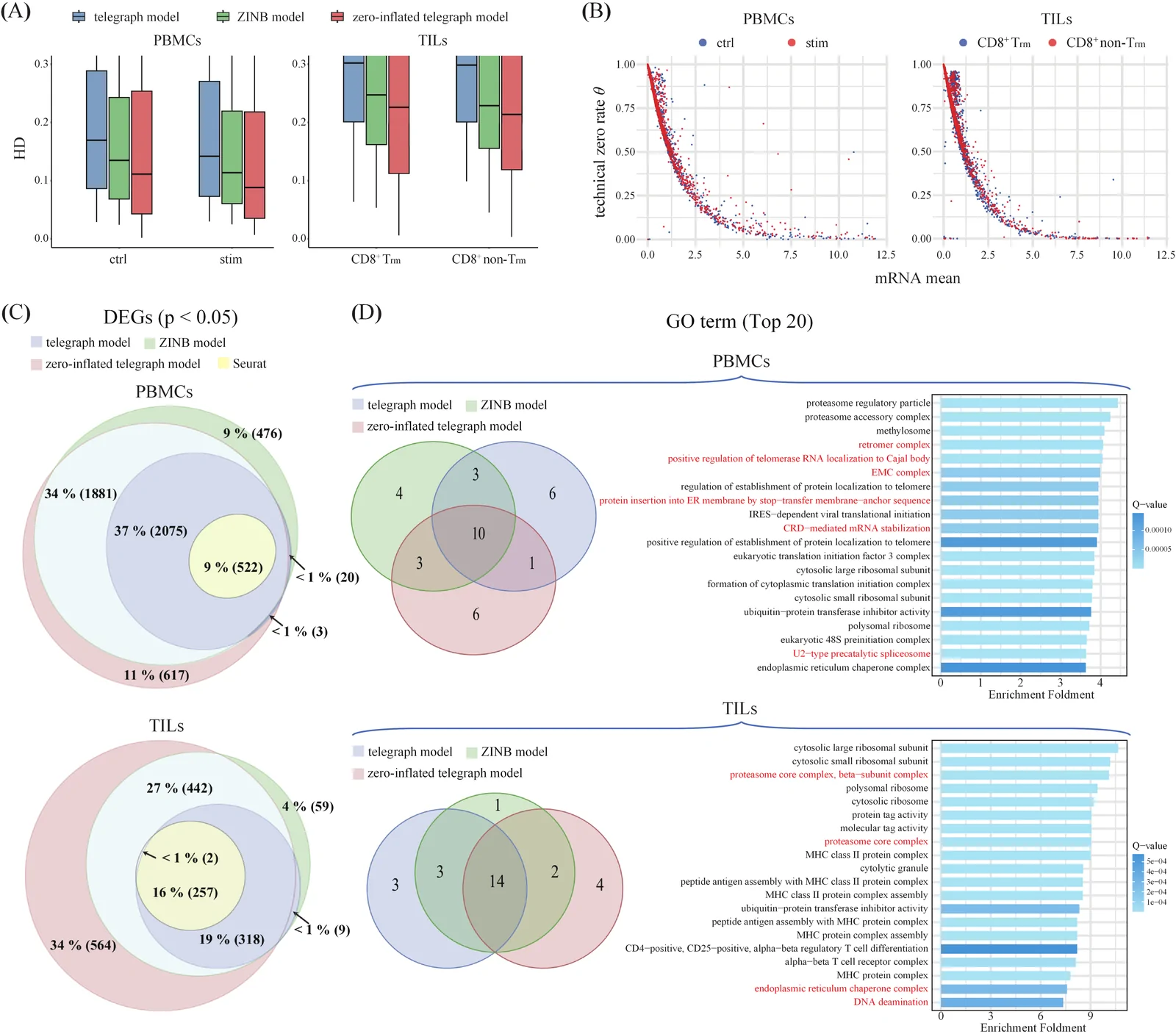
Zero-inflated telegraph model validation in human scRNA-seq transcriptomic data from lupus patient PBMCs  [31] and breast cancer patient TILs  [32]. **(A)** The zero-inflated telegraph model shows lower HD (better fitting) for mRNA distributions than the telegraph model and ZINB model. **(B)** The technical zero rate θ decreases with increasing mRNA mean for both datasets. **(C)** The zero-inflated telegraph model detects more DEGs than Seurat, the telegraph model, and the ZINB model, with notable advantages in small TIL samples. **(D)** Among the top 20 GO terms of the zero-inflated telegraph model, its DEGs share 14-16 GO terms with other models (marked in black), alongside unique terms (marked in red).

We compared DEGs analysis results from the three models and the conventional Seurat algorithm across PBMC and TIL datasets. As shown in Fig 6C, Seurat identified the fewest DEGs, while the telegraph model detected more than Seurat, and the two zero-inflated models yielded the most. Notably, in PBMC datasets (2,765 and 2,931 cells), the zero-inflated telegraph model identified slightly more DEGs than the ZINB model; in TIL datasets (606 and 1,097 cells), it detected significantly more. This aligns with Fig 5: the two zero-inflated models perform similarly with larger samples, but the zero-inflated telegraph model outperforms in small samples. Among unique DEGs of the zero-inflated telegraph model: In PBMCs, *LDLR* participates in low-density lipoprotein (LDL) metabolism, and its functional impairment causes plasma LDL accumulation, further inducing atherosclerosis-a common SLE comorbidity  [41]; *GZMB*, a CD8^+^ T cell marker, associates with cytotoxicity and interferon signatures, key in lupus immune dysregulation  [42]. In TILs, *FAIM2*, downregulated in tumors, links to immune infiltration and prognosis in breast cancer  [43]; *VDR* regulates immune modulation, critical for breast cancer microenvironment  [44]; *CLSPN*, a DNA damage response protein, promotes tumorigenesis via genomic instability, central to breast cancer progression  [45].

Finally, we performed GO term enrichment analysis on DEGs identified by the three models in the PBMC and TIL datasets (Fig 6D and Fig E in S1 Appendix). Notably, 14 of the top 20 GO terms from the zero-inflated telegraph model overlapped with the top 20 terms of at least one other model in PBMCs, while this overlap reached 16 in TILs (Fig 6D), confirming that the model retains its advantage of identifying more DEGs without compromising functional enrichment consistency. These overlapping terms align with the research focus: in PBMCs, shared terms include multiple proteasome-related functions, involved in antigen processing/presentation, cellular stress responses, and signal regulation, closely associated with SLE pathogenesis  [46,47]. In TILs, conserved terms include multiple ribosome-related functions, a process closely associated with breast cancer progression  [48]. Beyond these shared functions, the zero-inflated telegraph model also enriched for unique GO terms not detected by the other models. For example, in PBMCs, “U2-type precatalytic spliceosome” is linked to SLE, as the spliceosome autoantigen heterogeneous nuclear ribonucleoprotein A2 serves as a major T cell autoantigen in SLE patients  [49]. In TILs, “endoplasmic reticulum chaperone complex” is associated with breast cancer via its involvement in protein folding-related genes  [50], while “proteasome core complex” enhances the efficacy of proteasome inhibitors to activate CD8^+^ T cell-mediated anti-tumor immunity in breast cancer  [51].

### Zero-inflated telegraph model quantifies technical zeros in scRNA-seq data from mouse cells.

Here, we investigated whether the zero-inflated telegraph model can improve the description of scRNA-seq data. We selected three bimodal mRNA distributions: the C57 and CAST alleles of *Mbln2* gene in mouse fibroblasts  [11], and the P${\;}_{lac/ara}$ promoter in *E. coli*  [2]. Both the telegraph model and zero-inflated telegraph model were employed to fit these distributions. As shown in Fig 7A, the telegraph model failed to accurately capture the bimodality of all three distribution data; it incorrectly fitted the C57 and CAST alleles of *Mbln2* as unimodal distributions with non-zero peaks. A consistent limitation of the telegraph model fits was that the zero count in the fitted distribution was lower than the actual zero count in the data, suggesting the presence of notable technical zeros in the datasets. We therefore applied the zero-inflated telegraph model to these distributions, and it exhibited good agreement with the bimodal patterns, accurately matching both peaks; see Fig 7A. Incorporating zero inflation into the model thus significantly improves the fitting of bimodal mRNA distribution data.

**Fig 7:**
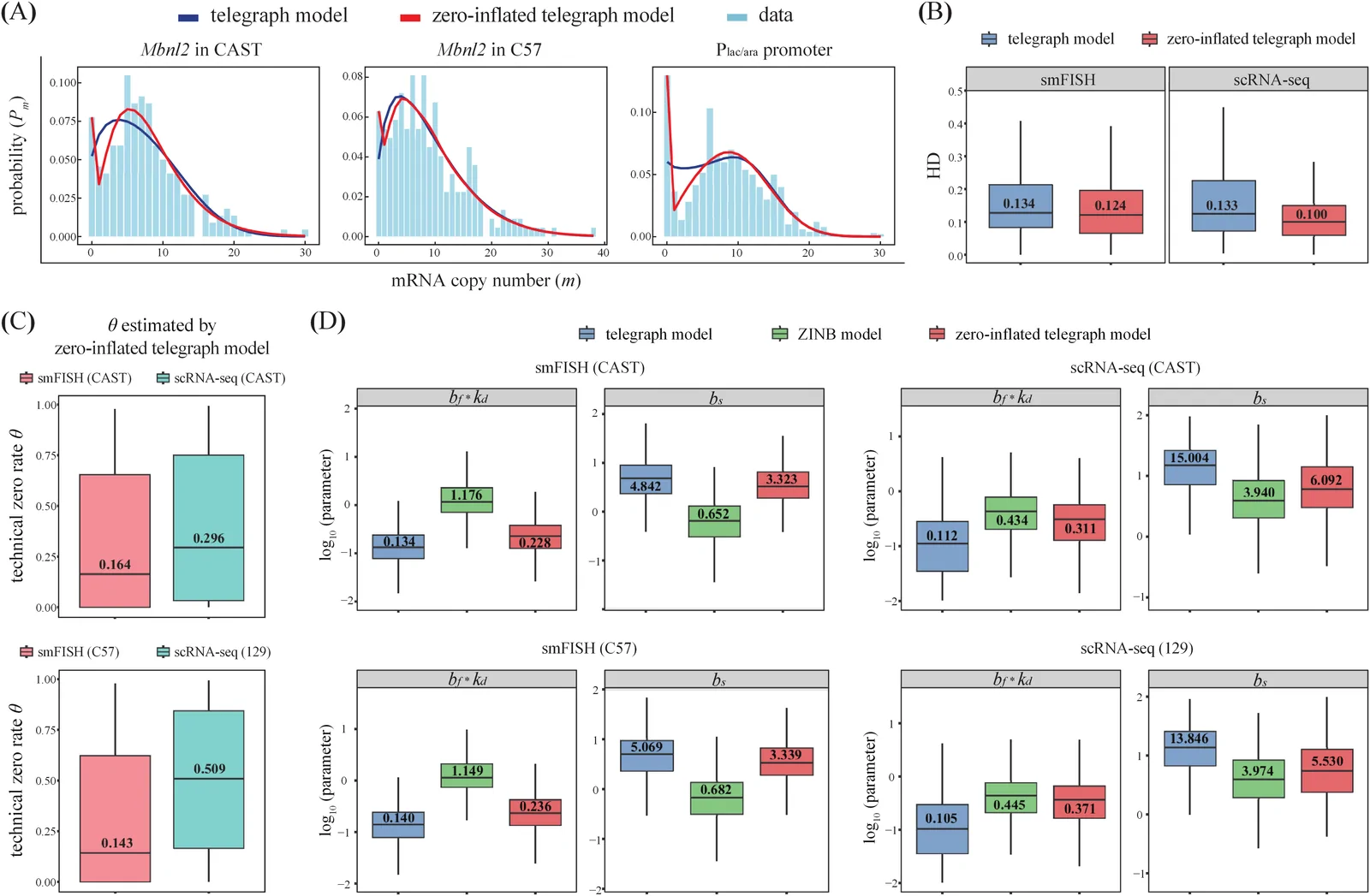
Performance comparison of zero-inflated telegraph model across two scRNA-seq protocols. **(A)** For single-cell transcription data (mouse fibroblasts *Mbln2* C57/CAST alleles  [11] and ***E.*** *coli* P${\;}_{lac/ara}$ promoter  [2]), the telegraph model misfits bimodal mRNA distributions as unimodal, while the zero-inflated telegraph model accurately captures bimodality. **(B)** For mouse ES cells genome-wide scRNA-seq data (Smart-seq2 and RamDA-seq)  [11,17], the zero-inflated telegraph model shows lower HD (better data fitting) than the telegraph model, with smaller HD differences in Smart-seq2 data. **(C)** The technical zero rate θ estimated by the zero-inflated telegraph model is lower in Smart-seq2 data (median θ = 0.14-0.16) than in RamDA-seq data (median θ = 0.3-0.5). **(D)** Relative to the zero-inflated telegraph model, the telegraph model underestimates $b_{f}$ and overestimates $b_{s}$, while the ZINB model shows the opposite bias. Compared with RamDA-seq data, the zero-inflated telegraph model has smaller differences in the estimated $b_{f}$ and $b_{s}$ with the telegraph model in Smart-seq2, but larger differences in these parameters with the ZINB model.

To further investigate the impact of zero inflation on scRNA-seq data fitting, we analyzed genome-wide CAST allele mRNA distribution data from mouse embryonic stem (ES) cells using two distinct single-cell sequencing strategies: a Smart-seq2 dataset covering 9,895 genes across 188 cells  [11], and a RamDA-seq dataset comprising 9,182 genes quantified over 419 cells  [17]. For rigorous comparison, we focused on the 1,988 genes shared between the two datasets. We fitted both datasets using the telegraph model and its extended zero-inflated variant. As anticipated, Fig 7B demonstrates that the zero-inflated telegraph model yielded lower HD values (better fitting performance) for both datasets. Notably, the difference in HD values between the two models was smaller for Smart-seq2 data than for RamDA-seq data, suggesting that Smart-seq2 data fitting is less affected by dropout events. This aligns with previous reports demonstrating that Smart-seq outperforms RamDA-seq for low-input RNA samples  [52]. To validate this observation, we estimated the technical zero rate θ from the zero-inflated telegraph model. As shown in Fig 7C, Smart-seq2 data exhibited substantially lower θ values: its median θ was at 0.16, compared to 0.3 for RamDA-seq data. We further validated the above trend using a mouse ES cell dataset containing C57 and 129 alleles. This dataset includes 958 shared genes profiled by Smart-seq2 for the C57 allele (188 cells)  [11] and by RamDA-seq for the 129 allele (419 cells)  [17]. The median θ was 0.14 for Smart-seq2 and 0.51 for RamDA-seq; see Fig 7C. Overall, parameter estimates from these two independent datasets demonstrate that the technical dropout rate θ is consistently lower for Smart-seq2 than for RamDA-seq.

Finally, we compared burst frequency $b_{f}$ and burst size $b_{s}$ across gene sets derived from mouse ES cells: 1,988 shared CAST allele genes, and 958 shared genes covering both C57 and 129 alleles. Parameters were inferred from Smart-seq2 and RamDA-seq data using the telegraph model, ZINB model and zero-inflated telegraph model. For each model and dataset type, we retained only genes with estimated $b_{f}$ and $b_{s}$ values within [0.01,100], thereby excluding extreme outliers without biological significance  [11,53]. We subsequently analyzed the distribution of these retained parameters; see Fig 7D. We note that the inferred $b_{f}$ corresponds to the ratio of the true $b_{f}$ to the degradation rate *k*_d_. We thus multiplied the inferred $b_{f}$ by the experimentally measured *k*_d_ from mouse cell data  [54] to obtain the actual value of $b_{f}$. For the CAST allele, compared with the standard telegraph model, the zero-inflated telegraph model yielded distinct parameter estimates: the standard telegraph model produced lower $b_{f}k_{\text{d}}$ (below the medians of 0.09 for Smart-seq2 and 0.2 for RamDA-seq data) and higher $b_{s}$ (above the medians of 1.54 for Smart-seq2 and 8.91 for RamDA-seq data). In contrast, the ZINB model gave larger $b_{f}k_{\text{d}}$ (above the medians of 0.95 for Smart-seq2 and 0.12 for RamDA-seq data) and smaller $b_{s}$ (below the medians of 2.65 for Smart-seq2 and 2.15 for RamDA-seq data). These results are consistent with the trends shown in Fig 2A. Comparable quantitative discrepancies were also observed for the C57 and 129 alleles (Fig 7D). Notably, differences in $b_{f}k_{\text{d}}$ (and $b_{s}$) between the telegraph model and zero-inflated telegraph model were less pronounced in Smart-seq2 data than in RamDA-seq data, indicating that the higher technical zero rate in RamDA-seq compromises the parameter accuracy of the telegraph model. In contrast, such differences were more striking between the ZINB model and zero-inflated telegraph model in Smart-seq2 data, indicating that the lower technical zero rate in Smart-seq2 highlights the need for more detailed gene regulatory mechanisms in model fitting.

## Conclusions and discussions

In this study, we addressed a critical gap in single-cell transcriptional data analysis by bridging zero inflation correction and biophysical transcriptional modeling. Conventional zero-inflated models correct for dropout but rely on unimodal distributions  [23], thus failing to capture transcriptional bursting-driven bimodality, a key feature of stochastic gene regulation  [1,12]. The classical telegraph model describes stochastic gene state-switching dynamics yet conflates biological and technical zeros. Notably, its more complex variants suffer from low computational efficiency  [55,56], while being sensitive to small sample sizes, extrinsic noise, and overfitting-all of which introduce biases into model selection and parameter inference  [20,57,58].

To this end, we developed the zero-inflated telegraph model, a robust framework that integrates technical zero correction with the classical telegraph model’s inherent ability to capture stochastic transcriptional bursting dynamics. Through systematic validation across diverse datasets, including synthetic data, human scRNA-seq data from lupus patient PBMCs  [31] and breast cancer TILs  [32], as well as mouse scRNA-seq data from ES cells  [11,17], we establish the model’s superiority over conventional approaches. Notably, zero-inflated telegraph model stands out for its enhanced biological interpretability, reliable inference of transcriptional regulation parameters, comprehensive detection of DEGs, and high computational efficiency, making it a practical tool for single-cell transcriptomic analysis.

The zero-inflated telegraph model demonstrates enhanced biological interpretability, reflected in its ability to capture mRNA distribution patterns and stabilize model selection. First, it enables stable model selection: even with a small sample size (*N* = 500) and strong extrinsic noise (20%), its correct selection rate remains nearly 70% or higher (Fig 1B). This ratio is comparable to that of the classical telegraph model  [20,58], indicating that incorporating technical zeros does not compromise the performance of this more complex model. Second, compared with conventional models, it shows an essential quantitative difference in mRNA distribution characterization (Fig 1A): it not only accurately fits bimodal distributions in *E. coli* and mouse mRNA distribution data (where traditional models even misfit bimodal distributions as unimodal ones; Fig 7A) but also performs better when fitting genome-wide transcriptional data from human (Fig 6A) and mouse (Fig 7B) scRNA-seq data. Notably, the ZINB model outperforms the classical telegraph model, collectively highlighting that accounting for zero inflation is more crucial for fitting genome-wide transcriptomic data, while incorporating the inherent mechanism of gene state-switching further improves fitting efficacy.

The zero-inflated telegraph model enables reliable inference of transcriptional bursting parameters: burst frequency $b_{f}$ (standardized by degradation rate) and burst size $b_{s}$. For one thing, it maintains robust $b_{f}$ and $b_{s}$ estimation when analyzing data integrating gene state-switching and dropout, even with small sample sizes (e.g., *N* = 500) and high extrinsic noise (e.g., 20%); see Fig 2A and 2B and 2C. For another, it degrades to the corresponding simple model when fitting synthetic data without dropout or inherent gene state-switching regulation, thus preventing overfitting (Fig 3). Notably, the ZINB model overestimates $b_{f}$ and underestimates $b_{s}$, while the telegraph model shows the opposite bias (underestimating $b_{f}$ and overestimating $b_{s}$), a discrepancy that may explain the excessively large $b_{s}$ relative to $b_{f}$ inferred by the telegraph model in bacteria, yeast, and mammalian cells  [7,8,53]. For example, in contrast to the telegraph model, which estimates smaller $b_{f}\sim 0.96$ $\left(k_{\text{d}}=0.5\;{min}^{-1}\right)$ and larger $b_{s}\sim 12.5$ for the P${\;}_{lac}$ promoter under high-growth stimulation  [8], RNA imaging directly measures P${\;}_{lac}$-based promoter as having $b_{f}\sim 1.93\;(k_{\text{d}}=0.014\;{min}^{-1})$ and $b_{s}\sim 2.4$ under the similar conditions  [2] (S1 Appendix). At the genome-wide scale, in mouse ES cells scRNA-seq data, the zero-inflated telegraph model corrects parameter inferences from the the telegraph model (median $b_{f}k_{\text{d}}$: 0.09-0.27; median $b_{s}$: 1.5-9) and the ZINB model (median $b_{f}k_{\text{d}}$: 0.07-0.95; median $b_{s}$: 1.5-2.7) to more accurate values (Fig  7D).

The zero-inflated telegraph model also enables reliable inference of the technical zero rate θ: inferred θ values are nearly independent of model type, sample size variations, or extrinsic noise, and decline to zero in synthetic data without dropout events (Fig 2D and Fig 3B and 3D). Furthermore, we performed a sensitivity analysis of system parameters based on the first three moments. The parameter θ showed the highest sensitivity among all parameters (Fig  F in S1 Appendix). This result demonstrates that accounting for zero inflation is essential for model construction. Applying θ inference to genome-wide human scRNA-seq transcriptomic data reveals higher θ for lowly expressed genes and lower θ for highly expressed genes (Fig 6B). Notably, scRNA-seq typically exhibits low expression levels  [17]. Comparing θ across two scRNA-seq datasets of mouse cells reveals lower technical zero rates for the Smart-seq2 platform relative to RamDA-seq. Specifically, the median absolute differences in θ between the two platforms reached 0.14-0.37 in ES cells (Fig 7C). This leads to a key observation (Fig 7B and 7D): the telegraph model performs better in data fitting and estimation of $b_{f}$ and $b_{s}$ for Smart-seq2 data than for RamDA-seq data. This underscores the need for caution when using the telegraph model for scRNA-seq data; ideally, the zero-inflated telegraph model should be used to eliminate the impact of technical zeros.

We validated the generalization ability of the zero-inflated telegraph model using synthetic data generated from biologically more realistic three-state transcription model and genetic toggle switch circuit  [37,39]. Benchmarks on datasets with and without sequencing dropout artifacts show that our model outperforms the telegraph model and ZINB model in both distribution fitting accuracy and the estimation reliability of the technical zero rate θ, allowing effective discrimination between biological and technical zeros (Fig 4A and 4B and 4E). Furthermore, our model also yields substantially higher accuracy in estimating burst frequency $b_{f}$ and burst size $b_{s}$(Fig 4C and 4D). These results suggest that the zero-inflated telegraph model can better characterize both technical zeros and the intrinsic gene regulatory mechanisms.

As expected, the zero-inflated telegraph model cannot perfectly infer $b_{f}$ and $b_{s}$ for complex regulatory systems. Nevertheless, its relative kinetic changes faithfully suggest the transcriptional mechanistic variations of the three-state model (Fig 4D). This finding is also supported by previous studies. The telegraph model has reliably captured genome-wide single-cell transcriptional bursting in mouse ES cells, fibroblasts and human cardiomyocytes  [11,17–19]. For genes under complex regulatory circuits, relative changes in its estimated effective $b_{f}$ and $b_{s}$ reflect genuine regulatory alterations in native systems. For instance, burst kinetics derived from the telegraph model successfully identifies synthetic negative feedback regulation in human kidney cells  [20]. Recent evidence further demonstrates that shifts in effective $b_{f}$ or $b_{s}$ correspond to major changes in the true parameters of complex transcriptional mechanisms  [19]. Collectively, although the zero-inflated telegraph model fails to output absolute accurate values of bursting parameters, it remains informative for dissecting intrinsic regulatory mechanisms. These merits render it a robust tool to detect differentially regulated genes across cellular contexts.

The zero-inflated telegraph model exhibits superior performance in detecting DEGs, outperforming the classical telegraph model, ZINB model, and conventional algorithms like Seurat. When applied to synthetic datasets simulating DEGs, the model achieved the highest AUC across all sample sizes, with particularly more notable advantages at the smaller sample size (Fig 5). In validation with human datasets, including CD14^+^ monocytes from lupus patient PBMCs and CD8^+^ T cell subsets from breast cancer TILs, the zero-inflated telegraph model detected more DEGs than its counterparts (Fig 6C). Notably, it captured disease-relevant unique DEGs that were missed by other methods, e.g., *LDLR* (links to SLE-atherosclerosis) and *GZMB* (associates with cytotoxicity and interferon signatures) in lupus PBMCs; *FAIM2* (links to immune infiltration) and *VDR* (regulates tumor microenvironment) in breast cancer TILs. Functional enrichment analysis confirmed the model’s DEGs aligned with disease mechanisms (Fig 6D): 14–16 of its top 20 GO terms overlapped with those of other models, while it also uncovered unique terms, e.g., “U2-type precatalytic spliceosome” links to SLE autoantigens in PBMCs and “endoplasmic reticulum chaperone complex” is critical for breast cancer protein folding in TILs, which further validate its biological relevance.

KEGG enrichment analysis further validated the biological functions of the DEGs identified by the zero-inflated telegraph model. *LDLR* was significantly enriched in the hsa05417 lipid and atherosclerosis pathway ($p=8.8\times 10^{-7}$). As atherosclerosis is a prevalent cardiovascular complication in patients with SLE  [41], this result solidifies the association between aberrant *LDLR* expression and SLE-related vascular pathological lesions. In comparison, although *FAIM2* was not enriched in canonical KEGG pathways, our enrichment analysis of breast cancer datasets revealed its significant enrichment in the apoptotic module of the hsa04210 cell growth and death pathway ($p=2.2\times 10^{-4}$). Mechanistically, *FAIM2* acts as a key *Fas* apoptosis inhibitory molecule that specifically regulates *Fas*-mediated apoptotic signaling  [43]. Consistent with this function, *FAIM2* drives breast cancer progression by inhibiting tumor cell apoptosis. Its interacting and co-expressed genes are enriched in diverse apoptosis and immune signaling pathways, including the p53 and TNF signaling pathways, as well as natural killer cell-mediated cytotoxicity, highlighting the core function of *FAIM2* in modulating breast cancer apoptosis and sustaining tumor microenvironment homeostasis.

Notably, we simplify technical-dropout modeling with a unified global θ, which enables our model to maintain the high computational efficiency and numerical stability of the original telegraph model. Several alternative methods handle variable technical zeros without manually introducing zero-inflation parameters. One approach attributes missing reads to cell-specific Beta-distributed capture rates, where stochastic binomial downsampling intrinsically yields zero values without artificial zero inflation  [59,60]. A second method separates true biological zeros from technical sequencing dropouts by measuring spliced (mature) and unspliced (nascent) RNAs  [16], though standard scRNA-seq datasets do not provide such paired RNA quantification. As previous mechanistic studies suggested  [61,62], combining nascent and mature RNA measurements can further improve parameter estimation of the model. Joint analysis of nascent and mature RNA distinguishes transcriptional elongation from post-transcriptional decay, reducing systematic biases in $b_{f}$ and $b_{s}$ caused by fitting solely on mature mRNA data. Drawing on these strategies, we will incorporate gene- and cell-specific θ alongside paired nascent and mature RNA data in future model development.

A key limitation of this study is that the classical telegraph model fails to fully capture the quantitative features of more complex regulatory mechanisms. Accordingly, its inferred burst frequency and burst size are effective parameters rather than true microscopic biochemical rates, which requires cautious biological interpretation. Future work should address this by incorporating additional regulatory mechanisms (e.g., multiple gene states, crosstalk signaling pathways  [13,15]) into computationally scalable frameworks, potentially integrating machine learning with stochastic modeling to improve parameter inference efficiency  [56,63]. Another limitation is the exclusive focus on the steady-state data, so subsequent research ought to move beyond this by coupling transcriptional burst dynamics with time-resolved single-cell measurements  [2,64] to understand cell fate determination and pathological processes. Additionally, the model standardizes mRNA degradation rate for computational simplicity, overlooking its variations across genes or cell states  [11,17,18]. Future work should integrate gene-specific degradation kinetics to cover more comprehensive gene expression regulation layers and correct such biases.

## Methods

In the following, we prove that the definition (3) guarantees that for any given observed distribution data ${\bar{P}}_{m},m=0,1,\cdots$, there exists a unique parameter set $\left(k_{\text{on}},k_{\text{off}},k_{\text{b}},\theta \right)$ that generates the data. We proceed by contradiction. Suppose there exist two distinct parameter sets $\left(k_{\text{on,1}},k_{\text{off,1}},k_{\text{b,1}},\theta_{1}\right)$ and $\left(k_{\text{on,2}},k_{\text{off,2}},k_{\text{b,2}},\theta_{2}\right)$ that yield the identical observed distribution ${\bar{P}}_{m}$ for all m=0,1,⋯. Denote the distributions generated by the telegraph model with parameters $\left(k_{\text{on,1}},k_{\text{off,1}},k_{\text{b,1}}\right)$ and $\left(k_{\text{on,2}},k_{\text{off,2}},k_{\text{b,2}}\right)$ as $P_{m}(1)$ and $P_{m}(2)$, respectively. We first claim that $\theta_{1}\text{≠}\theta_{2}$. Otherwise, if $\theta_{1}=\theta_{2}$, then (3) yields $P_{m}(1)=P_{m}(2)$ for all m≥0. Since the parameters $\left(k_{\text{on}},k_{\text{off}},k_{\text{b}}\right)$ are uniquely determined by the first three moments of $P_{m}$  [34], we immediately obtain $\left(k_{\text{on,1}},k_{\text{off,1}},k_{\text{b,1}})=(k_{\text{on,2}},k_{\text{off,2}},k_{\text{b,2}}\right)$. This contradicts the assumption that the two parameter sets are distinct. Hence, $\theta_{1}\text{≠}\theta_{2}$. Given $\theta_{1}\text{≠}\theta_{2}$, the definition (3) yields the following relation between the two distributions:


$$\begin{array}{c} P_{m}(2)=aP_{m}(1),\;m\geq 1,\text{ where }a=\frac{1-\theta_{1}}{1-\theta_{2}}. \end{array}$$

Moreover, both distributions satisfy the model recurrence relations  [4]:


$$\begin{array}{c} m(m+1)P_{m+1}(1)-m(k_{\text{on,1}}+k_{\text{off,1}}+k_{\text{b,1}}+m-1)P_{m}(1)+k_{\text{b,1}}(k_{\text{on,1}}+m-1)P_{m-1}(1)=0, \end{array}$$

$$\begin{array}{c} m(m+1)P_{m+1}(2)-m(k_{\text{on,2}}+k_{\text{off,2}}+k_{\text{b,2}}+m-1)P_{m}(2)+k_{\text{b,2}}(k_{\text{on,2}}+m-1)P_{m-1}(2)=0. \end{array}$$

For all m≥2, substitute $P_{m}(2)=aP_{m}(1)$, $P_{m+1}(2)=aP_{m+1}(1)$ and $P_{m-1}(2)=aP_{m-1}(1)$ into the above two recurrence equations and subtract the resulting equations. The common scaling factor *a* cancels out, leading to


$$\begin{array}{c} -m(k_{\text{on,1}}-k_{\text{on,2}}+k_{\text{off,1}}-k_{\text{off,2}}+k_{\text{b,1}}-k_{\text{b,2}})P_{m}(1)+[k_{\text{b,1}}(k_{\text{on,1}}+m-1)-k_{\text{b,2}}(k_{\text{on,2}}+m-1)]P_{m-1}(1)=0. \end{array}$$

Rearranging terms gives the ratio of successive distribution terms:


$$\begin{array}{c} \frac{P_{m}(1)}{P_{m-1}(1)}=\frac{k_{\text{b,1}}(k_{\text{on,1}}+m-1)-k_{\text{b,2}}(k_{\text{on,2}}+m-1)}{m(k_{\text{on,1}}-k_{\text{on,2}}+k_{\text{off,1}}-k_{\text{off,2}}+k_{\text{b,1}}-k_{\text{b,2}})}. \end{array}$$ (4)

We now show that $k_{\text{b,1}}=k_{\text{b,2}}$. Suppose, for contradiction, that $k_{\text{b,1}}\text{≠}k_{\text{b,2}}$. Taking the limit of (4) as m→∞, we obtain


$$\begin{array}{c} \lim_{m\to \infty }\frac{P_{m}(1)}{P_{m-1}(1)}=\frac{k_{\text{b,1}}-k_{\text{b,2}}}{k_{\text{on,1}}-k_{\text{on,2}}+k_{\text{off,1}}-k_{\text{off,2}}+k_{\text{b,1}}-k_{\text{b,2}}}. \end{array}$$ (5)

However, from the explicit integral representation (1) of the distribution, we have


$$\begin{array}{c} \frac{P_{m}(1)}{P_{m-1}(1)}=\frac{k_{\text{b,1}}}{m}\frac{\int_{0}^{1}e^{-k_{\text{b,1}}s}s^{k_{\text{on,1}}+m-1}(1-s{)}^{k_{\text{off,1}}-1}ds}{\int_{0}^{1}e^{-k_{\text{b,1}}s}s^{k_{\text{on,1}}+m-2}(1-s{)}^{k_{\text{off,1}}-1}ds}<\frac{k_{\text{b,1}}}{m}. \end{array}$$ (6)

As m→∞, the right-hand side tends to 0, which contradicts the existence of a finite non-zero limit (5). Therefore, $k_{\text{b,1}}=k_{\text{b,2}}$ must hold.

Substituting $k_{\text{b,1}}=k_{\text{b,2}}$ into the ratio formula (4) yields


$$\begin{array}{c} \frac{P_{m}(1)}{P_{m-1}(1)}=\frac{k_{\text{b,1}}}{m}\cdot \frac{k_{\text{on,1}}-k_{\text{on,2}}}{k_{\text{on,1}}-k_{\text{on,2}}+k_{\text{off,1}}-k_{\text{off,2}}}. \end{array}$$

Combining this result with the integral bound (6), we arrive at


$$\begin{array}{c} \frac{\int_{0}^{1}e^{-k_{\text{b,1}}s}s^{k_{\text{on,1}}+m-1}(1-s{)}^{k_{\text{off,1}}-1}ds}{\int_{0}^{1}e^{-k_{\text{b}}s}s^{k_{\text{on,1}}+m-2}(1-s{)}^{k_{\text{off,1}}-1}ds}=\frac{k_{\text{on,1}}-k_{\text{on,2}}}{k_{\text{on,1}}-k_{\text{on,2}}+k_{\text{off,1}}-k_{\text{off,2}}}. \end{array}$$

This result is impossible, as the left-hand side varies with *m*, while the right-hand side does not. Consequently, the assumption that two distinct parameter sets exist is invalid, which completes the proof of parameter uniqueness.

## Code accessibility

For the analyses presented in this study, the R code implementing the zero-inflated telegraph model, including mRNA distribution calculation, data fitting, parameter estimation and DEGs detection, is publicly available at https://github.com/Always-Stude/ZITM.git. This repository also contains scripts to generate synthetic datasets for both zero-inflated and standard model configurations.

## Supporting information

S1 AppendixFig A:Estimation of $b_{f}$ and $b_{s}$ under varying sample sizes and extrinsic noise. Synthetic data with dropouts were generated at *N* = 500, 1000, 2000 (complementing the *N* = 4000 results in Fig 2A and 2B). Estimation performance was compared among the telegraph model, ZINB model and zero-inflated telegraph model. **Fig B:** Correlation between the estimated technical zero rate θ and absolute relative errors of $b_{f}$ and $b_{s}$. A weak positive correlation was found between parameter errors and θ, with PCC values between 0.1 and 0.4. **Fig C:** Model performance on synthetic data from the telegraph model and ZINB model. The zero-inflated telegraph model provides robust parameter estimates for both types of datasets, while the other two models present distinct estimation biases. **Fig D:** Burst parameter inference on synthetic dropout-free data from the three-state transcription model. The telegraph model and zero-inflated telegraph model show similar accuracy, both outperforming the ZINB model. **Fig E:** GO enrichment analysis of DEGs from lupus and breast cancer datasets. Shared and unique enriched GO terms are marked in black and red, respectively. **Fig F:** Sensitivity analysis of the zero-inflated telegraph model parameters using distribution moments. The technical zero rate θ is the most sensitive parameter, highlighting the importance of zero-inflation correction.(PDF)
