# Supplementary material for: Integrating zero-inflation correction and transcriptional kinetics for single-cell transcriptomic analysis
Source: PLoS Comput Biol. 2026 Sep 25;22(9):e1014779. doi: 10.1371/journal.pcbi.1014779 (PMC13630325; doi:10.1371/journal.pcbi.1014779)
Supplement: Supplementary file 1 [file pcbi.1014779.s001.pdf]

S1 Appendix —
Integrating zero-inflation correction and transcriptional kinetics for single-cell transcriptomic analysis

Chengkai Yang[1,&], Yu Liao[1,&], Ying Sheng[1], Feng Jiao[1,2*]
[1] College of Mathematics and Information Sciences, Guangzhou University, Guangzhou, P. R. China
[2] Guangzhou Center for Applied Mathematics, Guangzhou University, Guangzhou, P. R. China
[&]C. Yang and Y. Liao contributed equally. [*] Correspondence: F. Jiao (jiaof@gzhu.edu.cn)

Contents

1 Introducing dropout events into synthetic mRNA distribution data **2**

2 Maximum-likelihood estimation method for model fitting and parameter inference **2**

3 Model selection based on AICc **2**

4 DEGs detection pipeline for the zero-inflated telegraph model **2**
4.1 Standardized method 2
4.2 Distribution computation of the model 3
4.3 DEGs analysis 3

5 Transcriptional bursting parameters for the P_{lac} promoter in *E. coli* **3**

6 Figures **5**

1 Introducing dropout events into synthetic mRNA distribution data

We employed the dropout model to introduce dropout events into the simulated data [1]. Specifically, let x_{ij} represent the synthetic expression level of gene i in cell j, and let μ denote the mean of non-zero expression levels (log read counts) of gene i across all cells. The dropout rate of gene i was modeled as $p_0 = e^{-\lambda\mu^2}$, where λ denotes the exponential decay parameter shared across all genes. We set $\lambda = 0.1$ in this study to generate synthetic data with dropout events that follow the desired distribution [1]. Subsequently, the expression level y_{ij} of gene i in cell j after introducing dropout events is defined as

$$y_{ij} = \begin{cases} x_{ij}, & \text{if } h_{ij} = 0, \\ 0, & \text{if } h_{ij} = 1, \end{cases} \quad \text{where} \quad h_{ij} \mid x_{ij} \sim \text{Bernoulli}(p_0).$$

2 Maximum-likelihood estimation method for model fitting and parameter inference

For each of the three models (telegraph model, ZINB model and zero-inflated telegraph model), we minimized the negative log-likelihood function, defined as [2–4]:

$$-\log L(\theta) = -\sum_m M(m) \log(P_m(\theta)), \tag{1}$$

where θ denotes the parameter set, $M(m)$ is the number of cells containing m copies of the transcript, and $P_m(\theta)$ represents the transcript distribution parameterized by θ. A critical step in this optimization is the selection of initial values of system parameters. For each model, we conducted 10 independent minimization runs for each optimization process, using randomly generated initial parameters: $k_{\text{on}}, k_{\text{off}}, b_f \in (0, 5), k_{\text{b}} \in (5, 50)$, with $b_s = k_{\text{b}}/k_{\text{off}}$; additionally, θ was set to P_0 (i.e., the probability of zero transcripts in the data).

3 Model selection based on AICc

For experimental steady-state mRNA distributions, we fit the data to the telegraph model, ZINB model, and zero-inflated telegraph model separately by minimizing the negative log-likelihood function $-\log L(\theta)$ defined in (1). To identify the best model for explaining the distribution data, a common approach is to select the one with the lower corrected Akaike information criterion (AICc)-a metric that incorporates penalties for the number of parameters and sample size [4, 5]:

$$\text{AICc} = -2 \log L(\theta) + 2k + \frac{2k(k+1)}{N - k - 1}.$$

Here k is the number of model parameters ($k = 3$ for the telegraph model and ZINB model, $k = 4$ for the zero-inflated telegraph model) and N is the cell sample size.

4 DEGs detection pipeline for the zero-inflated telegraph model

4.1 Standardized method

Let n_{ij} denote the read counts of gene i in cell j, then the size factor of cell j is estimated by $\hat{s}_j = \underset{i}{median} \frac{n_{ij}}{\left(\prod_{v=1}^{m} n_{iv}\right)^{1/m}}$, where $n_{iv} \neq 0$, namely only the non-zero read counts of each gene are used. Finally the normalized read counts of gene i in cell j is calculated by $n_{ij}/\hat{s}_j$ [1].

4.2 Distribution computation of the model

Let P_m and $\bar{P}_m$ denote the probability mass functions of the mRNA copy number distribution corresponding to the telegraph model and zero-inflated telegraph model, respectively. Their relationship is then given by:

$$\bar{P}_0 = \frac{N_0 + \theta(N - N_0)}{N} = P_0 + \theta(1 - P_0), \quad \bar{P}_m = \frac{N_m - \theta N_m}{N} = (1 - \theta)P_m, \;\; m \geq 1.$$

Let P_m corresponding to the telegraph model be treated as a beta-Poisson distribution [6], in which case $\bar{P}_m$ is given by the following form:

$$\bar{P}_m = \begin{cases} \theta + \frac{(1-\theta)f}{\beta(k_{\text{on}},k_{\text{off}})\cdot 2^{1-k_{\text{on}}-k_{\text{off}}}}, & m = 0, \\ \frac{(1-\theta)f}{\beta(k_{\text{on}},k_{\text{off}})\cdot 2^{1-k_{\text{on}}-k_{\text{off}}}}, & \text{otherwise}, \end{cases}$$

where β denotes the Beta distribution, and f represents the hypergeometric function integral:

$$f = \frac{1}{m!}\int_{-1}^{1}\left(\frac{k_{\text{b}}(t+1)}{2}\right)^{m}(1-t)^{k_{\text{off}}-1}(1+t)^{k_{\text{on}}-1}e^{-\frac{k_{\text{b}}(t+1)}{2}}dt.$$

The integral can be easily and rapidly computed by using the Gauss-Jacobi quadrature method [6].

4.3 DEGs analysis

We incorporated the zero-inflated telegraph model into the generalized linear model (GLM) framework to perform DEGs analysis. In general, given response variables $Y = \{y_i\}_{i=1}^{n}$ and explanatory variables $X = \{x_j\}_{j=1}^{k}$, a GLM comprises a linear predictor

$$\eta_i = \beta_0 + \beta_1 x_{1i} + \cdots + \beta_k x_{ki},$$

here $\mu_i = E(y_i)$ denotes the expectation of the response variable y_i, and this expectation μ_i is linked to the linear predictor η_i via a specified link function.

In order to estimate the model parameters we use the iterative weighted least-squares (IWLS) algorithm [6], which only requires a variance function that specifies the relationship between the mean μ and variance *Var* of explanatory variables X, which is

$$Var(X) = \mu + \mu^2\left(\frac{k_{\text{off}}}{k_{\text{on}}\left(k_{\text{on}} + k_{\text{off}} + 1\right)}\right).$$

For between-group comparisons, we assume distinct group-specific means μ, and then utilize the glm($\cdot$) function in R to fit the GLM. The entire analytical procedure, encompassing differential expression analysis, is termed ZeroBP. This also serves as the name of the accompanying R package, which is publicly available at https://github.com/Always-Stude/ZeroBP.

5 Transcriptional bursting parameters for the P_{lac} promoter in *E. coli*

Two studies [7, 8] focused on *E. coli* as the host, with the P_{lac} promoter or its hybrid derivative $\text{P}_{\text{lac/ara}}$ (consistent with the regulatory logic of P_{lac}) as the target. To maximize transcriptional activity, high-growth conditions were standardized across experiments: 1 mM isopropyl β-D-thiogalactopyranoside was added to derepress the *LacI* repressor, relieving its inhibition on P_{lac} [7]; 10 mM cyclic AMP was supplemented to activate the cAMP receptor protein, a transcription factor that enhances P_{lac} transcriptional strength [7]; For the $\text{P}_{\text{lac/ara}}$ hybrid promoter [8], 0.1% arabinose was included to activate the AraC activator, ensuring full induction of the promoter.

For the $P_{lac/ara}$ promoter analyzed via MS2-GFP live-cell RNA imaging in [8], time-lapse microscopy revealed exponential distributions of transcriptional OFF and ON periods, with mean 37 min and 6 min, respectively. This gives $k_{\rm on} = 1/37$ min^{-1} and $k_{\rm off} = 1/6$ min^{-1}. Transcripts degradation rate $k_{\rm d} = 0.014 \pm 0.002$ min^{-1} (dominated by cell division), synthesis rate $k_{\rm b} = 1/2.5$ min^{-1} (2.5 min to transcribe 1 message during gene on state). Therefore, the standardized burst frequency $b_f = k_{\rm on}/k_{\rm d} \sim 1.93$ and burst size $b_s = k_{\rm b}/k_{\rm off} = 2.4$.

For the $P_{\rm lac}$ promoter analyzed via smFISH in [7], fitting mRNA mean M and variance to the telegraph model gave stable $k_{\rm on} \sim 0.48$ min^{-1} and $k_{\rm b} \sim 30$ min^{-1} across growth conditions. mRNA degradation rate $k_{\rm d} \sim 1/2$ (from measured ∼2 min lifetime). $k_{\rm off}$ varies in response to growth conditional change, and at the maximum growth stimulation, $k_{\rm off} \sim 2.4$ min^{-1} can be reversely calculated from the mean level $M = \frac{k_{\rm b} k_{\rm on}}{k_{\rm d}(k_{\rm on}+k_{\rm off})} \sim 10$. Therefore, the standardized burst parameters $b_f = k_{\rm b}/k_{\rm d} \sim 0.96$ and $b_s = k_{\rm b}/k_{\rm off} \sim 12.5$.

References

[1] Miao, Z., Deng, K., Wang, X. and Zhang, X. (2018) DEsingle for detecting three types of differential expression in single-cell RNA-seq data. *Bioinformatics* **34**, 3223-3224.

[2] Larsson, A.J.M. *et al.* (2019) Genomic encoding of transcriptional burst kinetics. *Nature* **565**, 251-254.

[3] Raj, A., Peskin, C.S., Tranchina, D., Vargas, D.Y. and Tyagi, S. (2006) Stochastic mRNA synthesis in mammalian cells. *PLoS Biol.* **4**, e309: 1707-1719.

[4] Jiao, F. *et al.* (2024) What can we learn when fitting a complex gene expression model to a simple telegraph? *PLoS Comput. Biol.* **20**, e1012118.

[5] Zhu, C. *et al.* (2025) Correlation and distinction between stochastic gene transcription models with and without polymerase dynamics. *Phys. Rev. Res.* **7**, 023050.

[6] Vu, T.N. *et al.* (2016) Beta-Poisson model for single-cell RNA-seq data analyses. *Bioinformatics* **32**, 2128-2135.

[7] So, L. *et al.* (2011) General properties of the transcriptional timeseries in Escherichia Coli. *Nat. Genet.* **43**, 554-560.

[8] Golding, I., Paulsson, J., Zawilski, S. and Cox, E. (2005) Real-time kinetics of gene activity in individual bacteria. *Cell* **123**, 1025-1036.

6 Figures

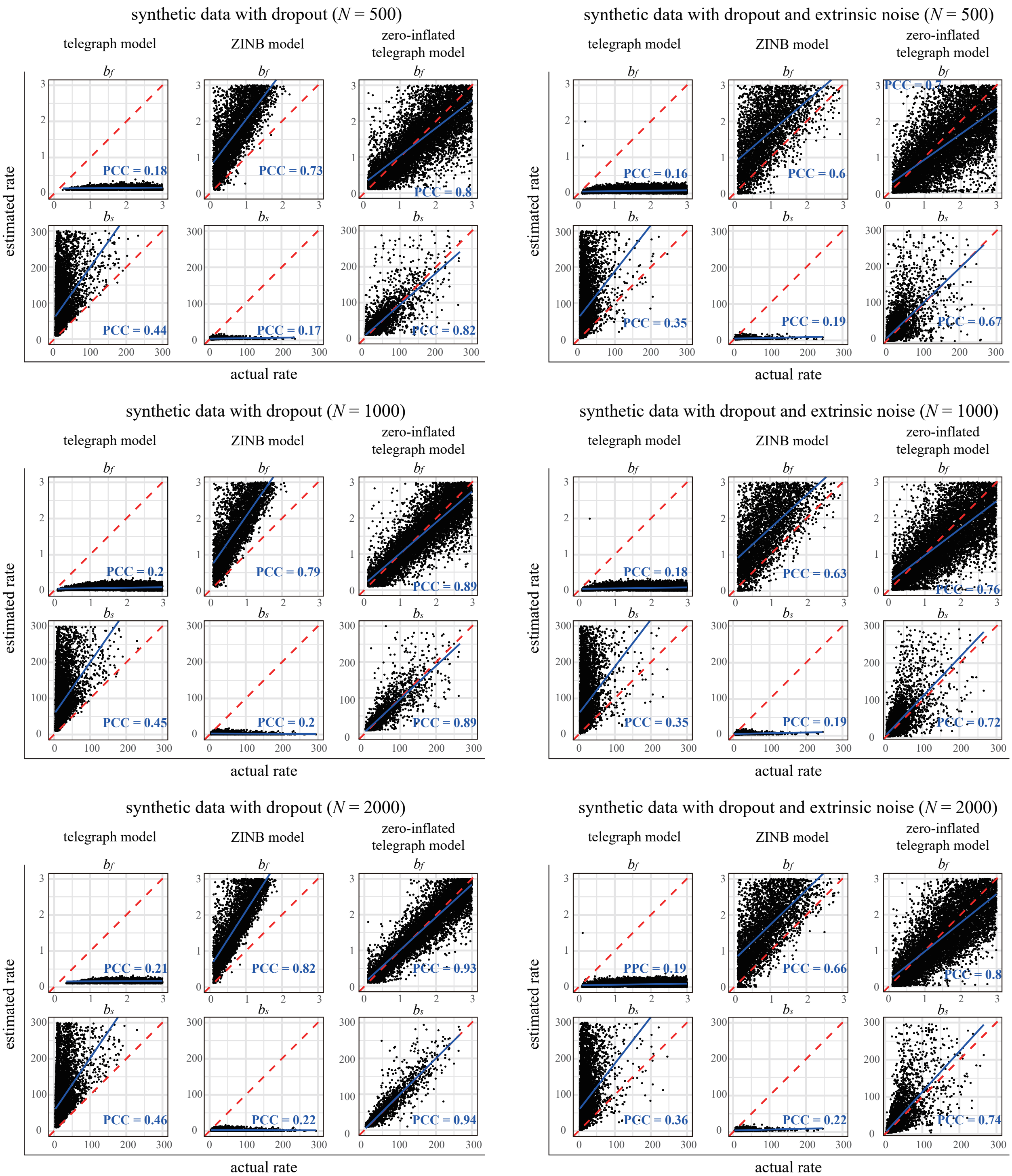

Fig A: **Transcriptional burst parameter estimation across different sample sizes and extrinsic noise conditions.** Synthetic distribution data with dropout events were generated via the telegraph model across sample sizes $N = 500, 1000, 2000$ (complementing the $N = 4000$ results in Fig. 2a,b) and with/without 20% extrinsic noise. Scatter plots comparing estimated vs. true values of transcriptional burst frequency b_f and burst size b_s for the telegraph model, ZINB model, and zero-inflated telegraph model.

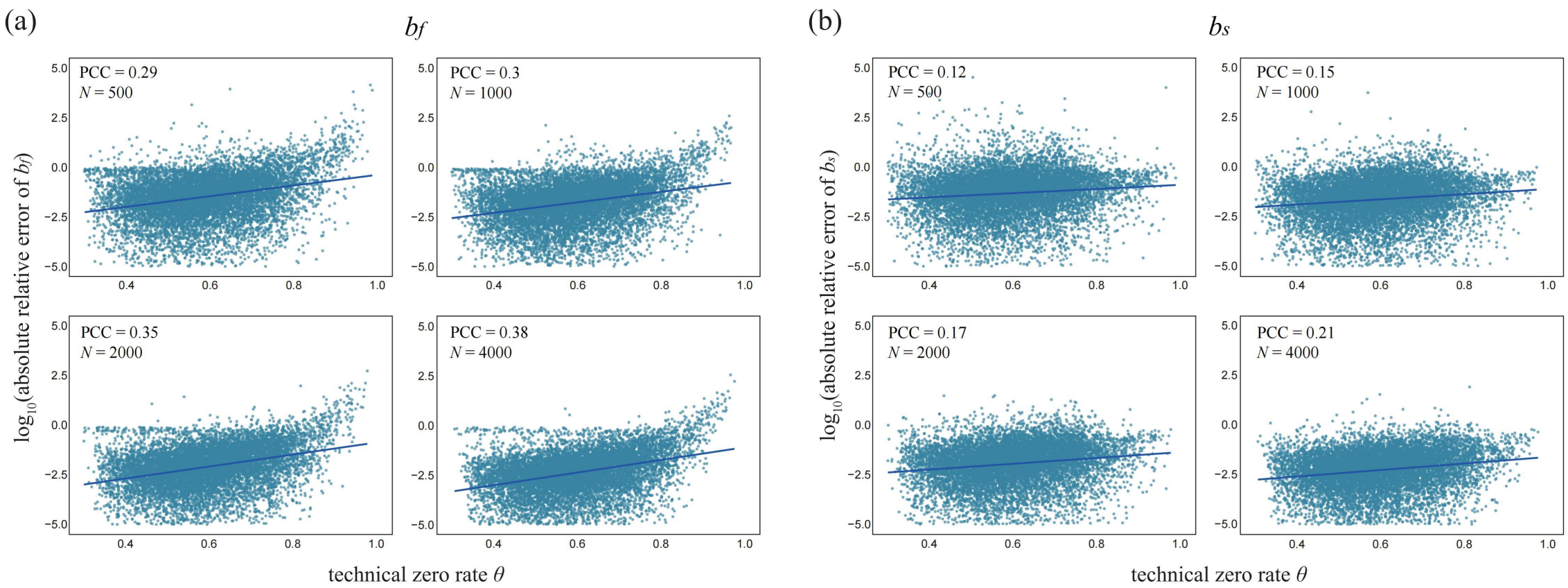

Fig B: **Correlation between estimated technical zero rate θ and relative errors of burst parameters.** Synthetic datasets with dropout events were generated via the telegraph model across sample sizes $N = 500, 1000, 2000, 4000$. We computed the absolute relative errors of inferred burst frequency b_f in (a) and burst size b_s in (b). A weak positive correlation was observed between these estimation errors and the estimated technical zero rate θ, with PCC ranging from 0.1 to 0.4.

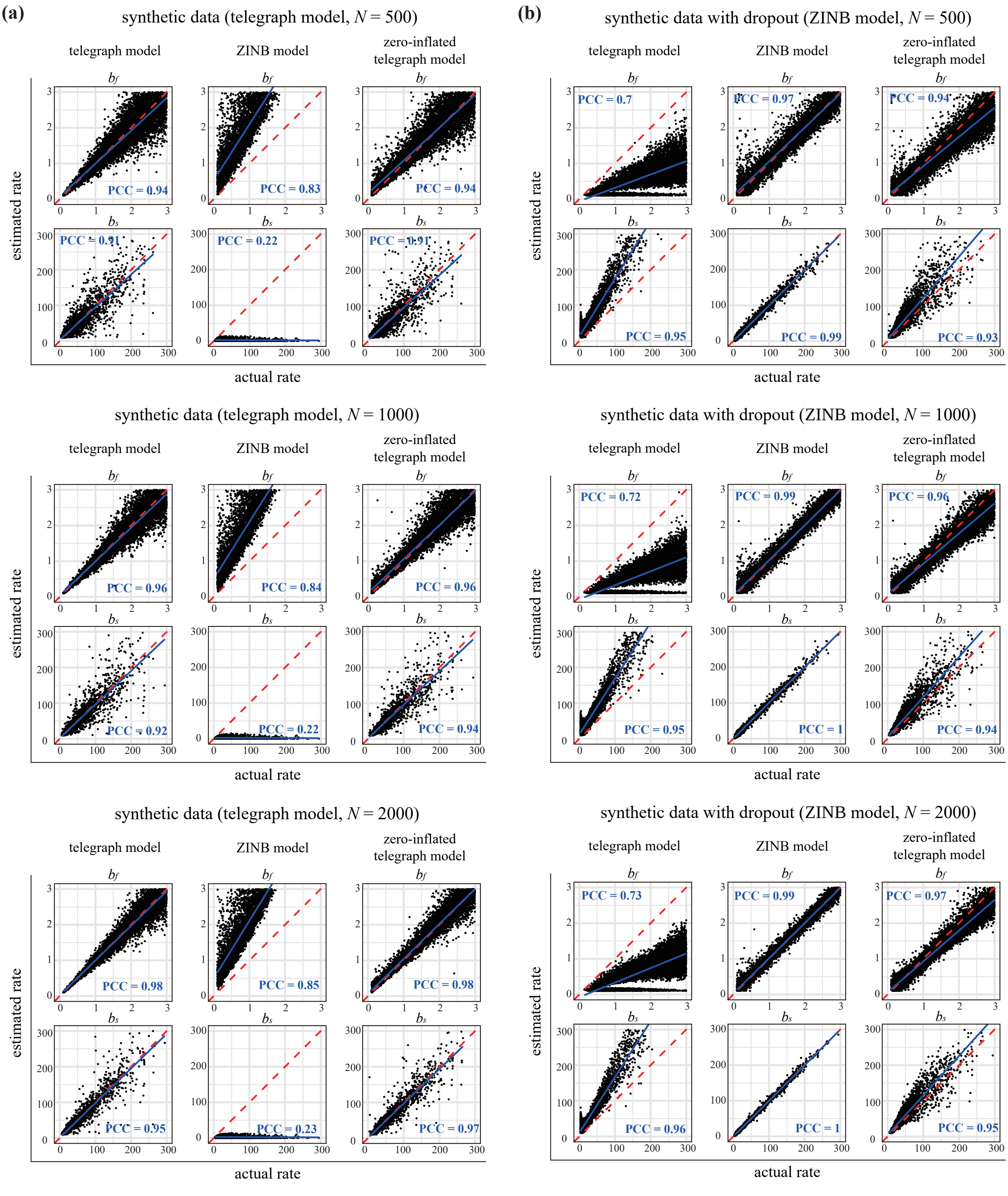

Fig C: **Zero-inflated telegraph model fitting simpler synthetic data.** **(a)** For telegraph model-generated data (sample sizes $N = 500, 2000, 2000$), the zero-inflated telegraph model's b_f and b_s estimates cluster tightly along the 1:1 reference line (PCC > 0.9), while the ZINB model shows obvious b_f and b_s estimation bias. **(b)** For bursty model-generated dropout-containing data (sample sizes $N = 500, 2000, 2000$), the telegraph model underestimates b_f and overestimates b_s, while both zero-inflated models give reliable b_f and b_s estimates (PCC > 0.9).

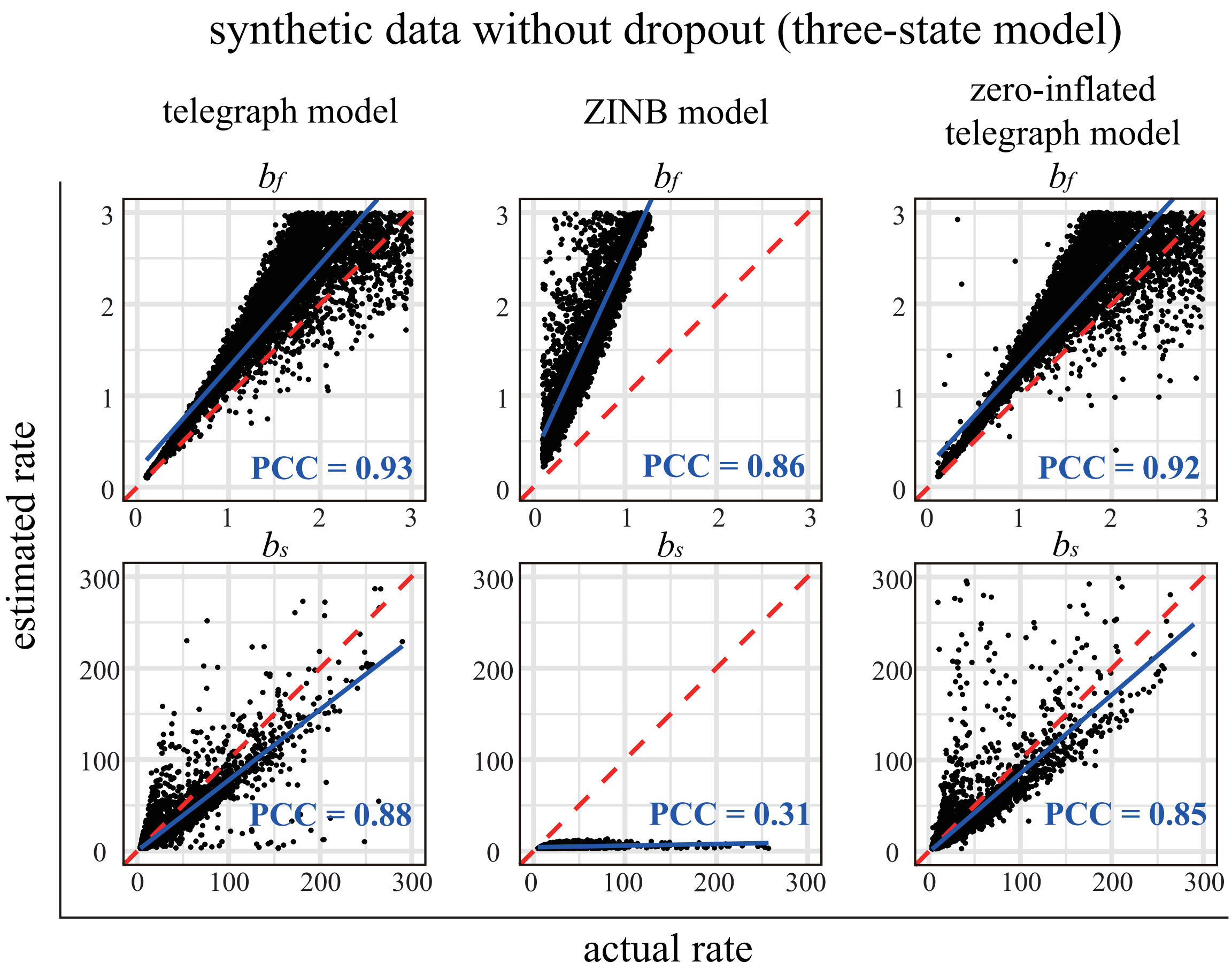

Fig D: **Estimation performance of burst parameters on dropout-free synthetic data from the three-state model.** Synthetic datasets without sequencing dropouts were generated using the three-state transcription model. We compared the inference accuracy of burst frequency b_f and burst size b_s across the telegraph model, ZINB model and zero-inflated telegraph model. The zero-inflated telegraph model achieves comparable performance to the classical telegraph model, and both outperform the ZINB model substantially.

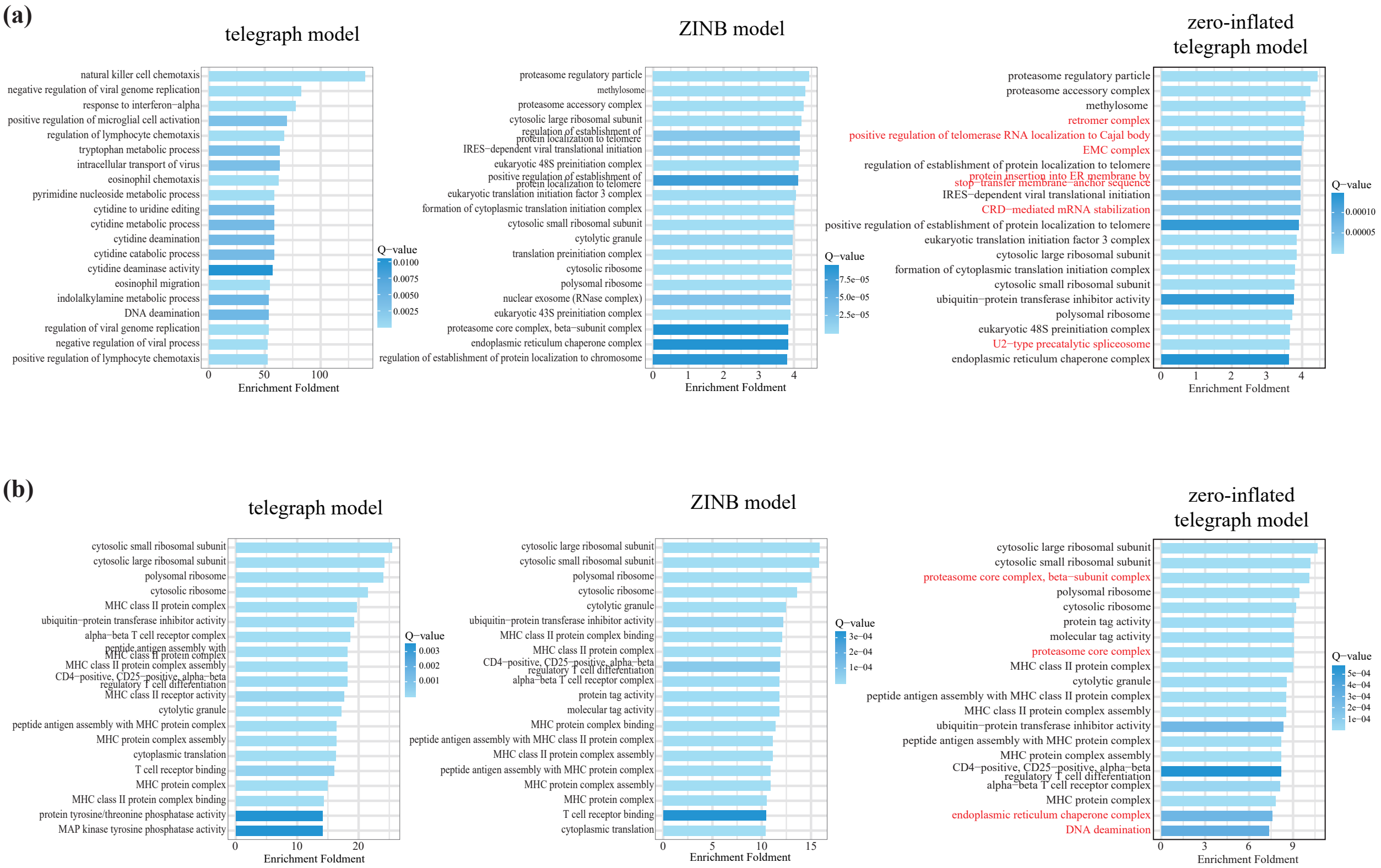

Fig E: **GO functional enrichment analysis of DEGs identified in lupus patient PBMC (a) and breast cancer patient TIL (b) datasets.** Detailed enrichment results were derived from DEGs detected by three models: the telegraph model, ZINB model, and zero-inflated telegraph model. Among the top 20 GO terms of the zero-inflated telegraph model, the DEGs identified by this model shared 14-16 terms with those from the other two models (marked in black), while also uncovered unique GO terms (marked in red).

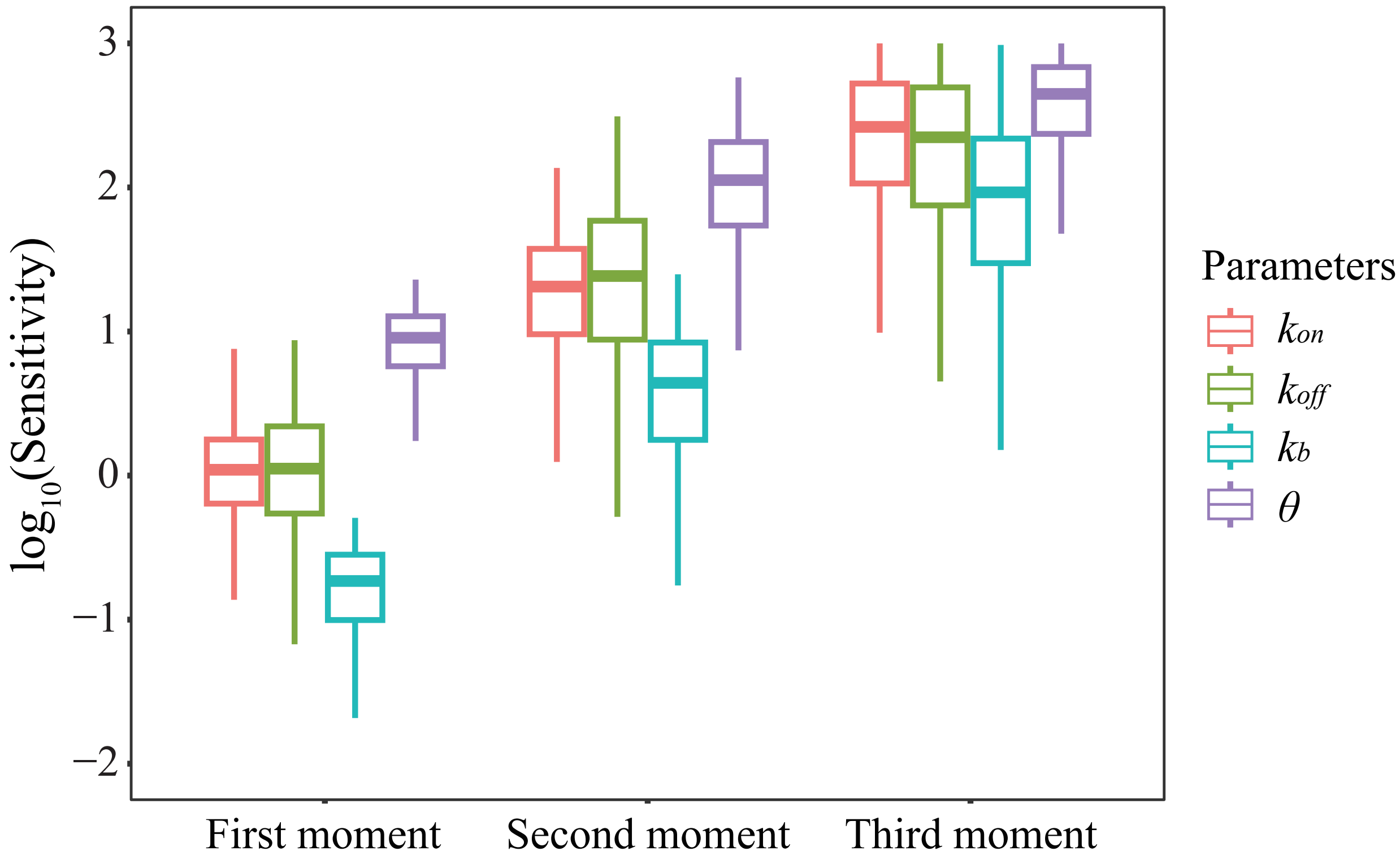

Fig F: **Sensitivity analysis of parameters for the zero-inflated telegraph model based on the first three moments.** We assessed the sensitivity of all parameters of the zero-inflated telegraph model using the first three moments of mRNA distributions. The technical zero rate θ showed the highest sensitivity, indicating that zero-inflation correction is indispensable for constructing a reliable model.
